# Validation of computational fluid dynamics of shake flask experiments at moderate viscosity by liquid distributions and volumetric power inputs

**DOI:** 10.1038/s41598-024-53980-7

**Published:** 2024-02-13

**Authors:** Carl Dinter, Andreas Gumprecht, Matthias Alexander Menze, Amizon Azizan, Paul-Joachim Niehoff, Sven Hansen, Jochen Büchs

**Affiliations:** 1 RWTH Aachen University, Forckenbeckstraße 51, 52074 Aachen, Germany; 2 Evonik Operations GmbH, Rodenbacher Chaussee 4, 63457 Hanau-Wolfgang, Germany; 3 School of Chemical Engineering, College of Engineering, Universiti Teknologi MARA, 40450 Shah Alam, Selangor Malaysia; 4 Evonik Operations GmbH, Paul-Baumann-Straße 1, 45772 Marl, Germany

**Keywords:** Chemical engineering, Chemical engineering, Biotechnology

## Abstract

Computational fluid dynamics (CFD) has recently become a pivotal tool in the design and scale-up of bioprocesses. While CFD has been extensively utilized for stirred tank reactors (STRs), there exists a relatively limited body of literature focusing on CFD applications for shake flasks, almost exclusively concentrated on fluids at waterlike viscosity. The importance of CFD model validation cannot be overstated. While techniques to elucidate the internal flow field are necessary for model validation in STRs, the liquid distribution, caused by the orbital shaking motion of shake flasks, can be exploited for model validation. An OpenFOAM CFD model for shake flasks has been established. Calculated liquid distributions were compared to suitable, previously published experimental data. Across a broad range of shaking conditions, at waterlike and moderate viscosity (16.7 mPa∙s), the CFD model's liquid distributions align excellently with the experimental data, in terms of overall shape and position of the liquid relative to the direction of the centrifugal force. Additionally, the CFD model was used to calculate the volumetric power input, based on the energy dissipation. Depending on the shaking conditions, the computed volumetric power inputs range from 0.1 to 7 kW/m^3^ and differed on average by 0.01 kW/m^3^ from measured literature data.

## Introduction

In biochemical engineering, shake flasks and stirred tank reactors (STRs) are two of the most frequently used reactor designs. Shake flasks are utilised in the early stages of process development and STRs in the later stages of process development and at production scale. Despite their frequent use, the scale-up from shake flasks to STRs remains challenging^1^. The most common scale-up parameters are the volumetric gas–liquid mass transfer rate (k_L_a) and the volumetric power input^2^.

CFD is already routinely used for STRs with great success to calculate mixing times and inhomogeneities, the k_L_a, gas hold-up, volumetric power input and shear stress^3–13^. In contrast, CFD applications for small scale, shaken bioreactors, such as microtiter plates^14–18^ and shake flasks^19–22^, are scarce in the scientific literature. Zhang et al. used a CFD model for shake flasks to calculate the volumetric power input and k_L_a for a variety of shaking parameters^19^. Similarly, Li et al. calculated the k_L_a value from CFD models, while investigating the influence of baffles in shake flask^20^. Furthermore, they determined the effect of shear stress on mycelial cell cultures. Liu et al. also used a CFD model to compute k_L_a and shear stress in baffled and unbaffled flasks, in order to investigate the effects on plant cell cultures^21^. In contrast to Li et al.^20^, they not only calculated average shear stress, but also proposed an approach to estimate the distribution of hydromechanical stress. To our knowledge, Mehmood et al. published the only CFD simulations in shake flasks with non-Newtonian, shear-thinning liquids. The effect of the shear-thinning behaviour is, however, minuscule. Above shear rates of 100 s^−1^, which is exceeded in virtually all shake flask experiments, the fluids show a Newtonian plateau, resulting in an elevated viscosity of up to 3 mPa∙s with essentially Newtonian behaviour^22^. They calculated power dissipation and k_L_a, assuming laminar flow, outlining the importance of these parameters for culture performance of a filamentous microorganism^22^. CFD models have also been already used for the development of novel shaken bioreactor designs^23,24^. For example, He et al. used a CFD model, employing a RNG k-epsilon turbulence model, to design a novel well type for microtiter plates, computing the k_L_a at a variety of shaking conditions, including viscosities of up to 30 mPa∙s^18^.

**Figure 1:**
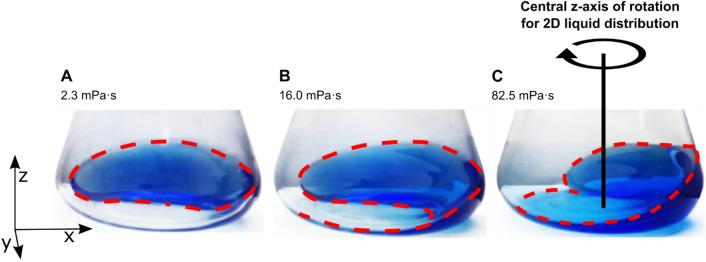
Liquid distribution as a function of viscosity (Image adapted from Sieben et al.^40^). Photographs of the rotating liquid in shake flasks are shown at (**A**) 2.3 mPa∙s, (**B**) 16 mPa∙s and (**C**) 82.5 mPa∙s, taken with a rotating camera, which is fixed, relative to the direction of centrifugal force and, hence, in a fixed position to the bulk liquid^35,36^. The centrifugal force is pointing in the negative y-direction in all 3 images. With the dashed red line, the liquid contact line at the intersection of liquid, glass and air is highlighted. This contact line is considered representative for the entire liquid distribution and responds to changes in the operating conditions, as shown for viscosity. The contact line is, therefore, an ideal tool to validate CFD calculations. Shaking conditions: Filling volume (V_L_) = 30 mL, shaking frequency (n) = 150 rpm, shaking diameter (d_0_) = 5 cm, temperature (T) = 25 °C.

A comprehensive validation of CFD models is crucial to achieve reliable results. In principle. two avenues for directly validating the liquid flow in shake flasks exist. Firstly, the internal fluid flow can be recorded with techniques like Particle Image Velocimetry^25–27^ (PIV) and Particle Tracking Velocimetry^28,29^ (PTV). Both techniques are the most common and accurate techniques to measure the internal liquid flow in STRs^6^. They have already been used before in the small scale, e.g., cylindrical bioreactors^30,31^ and shake flasks by Palacios-Morales et al.^32^. Palacios-Morales et al. provide a great inside into the flow behaviour in shake flask and investigate the effects of baffle structures of the fluid flow. They do so for a single set of experimental conditions at a relatively low shaking frequency of 150 rpm. Unfortunately, this does not provide enough data for thoroughly validating a CFD model for shake flasks. Secondly, the liquid flow in CFD models for shake flasks can be validated by the externally observable liquid distribution. This approach has already been used in the literature for the validation of larger, orbitally shaken, cylindrical bioreactors^33,34^. The orbital shaking motion in orbitally shaken experiments drives the liquid along the flask wall, leading to distinct, externally observable liquid distributions (see Fig. 1). In non-baffled shake flasks such liquid distributions are in a quasi-steady state, except for the angular rotation due to the shaking motion. Hence, CFD models for shake flasks can simply be validated by comparing the liquid distribution, taken from CFD simulations, to photographs of the rotating bulk liquid in shake flasks^19^. The necessary photographs can be obtained with a rotating camera, where the angular position relative to the centrifugal force and, hence, to the bulk liquid remains constant^35,36^. Although a direct comparison to photographs is possible, the approach is hardly quantifiable and highly subjective. Zhu et al. improved this approach by recording the liquid distribution of orbitally shaken cylinders with filling volumes of 2.5–10 L and smaller cylinders with a conical bottom with high-speed cameras, measuring the liquid height in those videos^33,34^.

Azizan et al. aimed to provide quantitative data for the liquid distributions in shake flasks^37,38^. Based on previous work by Ottow et al.^39^, the devised measurement system employed non-invasive fluorescence measurements. Liquid contact lines at the intersection of liquid, air and glass were recorded at 14 distinct heights, each 5 mm apart. Liquid contact lines are representative of the entire liquid distribution and are responsive to changes in the operating conditions. Illustrations of such liquid contact lines are depicted as dashed red lines in Fig. 1 for a variation of the viscosity. The overall shape of the liquid distribution and, consequently, the liquid contact line changes with the increase in viscosity. For all three viscosities, the centrifugal force points in the same direction, indicating the shaker tray is in the same angular position. The leading edge of the rotating bulk liquid is shifted against the shaking direction, out of alignment with the centrifugal force, as can be seen in Fig. 1. Sieben et al.^40^, Ladner et al.^41^ and Hoffmann et al.^42^ have previously exploited this shift of the leading edge against the shaking direction to measure the viscosity in shake flask experiments. Further, at a critical viscosity, the position of the bulk liquid will be entirely out-of-phase with the shaking motion, leading to a complete collapse of the liquid distribution. A detailed description of the out-of-phase phenomenon can be found in Büchs et al.^43,44^ and Azizan et al.^38^.

Previous publications, regarding CFD in shake flasks have almost exclusively focused on waterlike viscosity. Elevated viscosities are, however, commonly found in bioprocesses involving filamentous morphology or biopolymers^45^. Famous examples of filamentously growing organisms include the fungi *Penicillium chrysogenum* and *Aspergillus niger*, which produce antibiotics and citric acid, respectively^43–46^. Typical examples also include biopolymer producers, such as *Xanthomonas campestris* for xanthan^46,47^ and *Azotobacter vinelandii* for alginate^48–50^. Importantly, medium viscosity has a significant influence on mixing, gas–liquid mass transfer and volumetric power input, making it a crucial process parameter to consider in bioprocess design.

This work aims to establish and validate a CFD model for shake flasks for Newtonian fluids with waterlike and moderate viscosity. The model will be validated by experimental liquid distribution data from Azizan et al.^37,38^, in terms of the shape of the liquid distribution and overall position relative to the centrifugal force, at a variety of shaking conditions. Additionally, the volumetric power input will be calculated and compared to experimentally determined volumetric power inputs and the correlation from Büchs et al.^43,44^.

Prior to validating the CFD model by running a large number of simulations, the model was tuned for optimal performance on a small number of simulations. Firstly, the liquid contact lines, extracted from the CFD model were compared to the simplified mechanistic model assuming negligible viscosity, developed by Büchs et al.^51^. Secondly, the influence of the precise shake flask geometry on liquid contact lines at waterlike viscosity was investigated. Thirdly, a first simulation was performed at a moderate viscosity of 16.7 mPa∙s. It revealed the impact of the liquid films formed on the flask wall by the rotating bulk liquid on the extraction of liquid contact lines. Following the tuning of the CFD model, a large number of shaking conditions were simulated and extracted liquid contact lines compared to the experimental liquid contact lines determined by Azizan et al.^37^. After the validation, the CFD model was used to calculate volumetric power inputs for a number of shaking conditions.

## Results and discussion

### CFD model validation by comparison of liquid distributions

**Figure 2:**
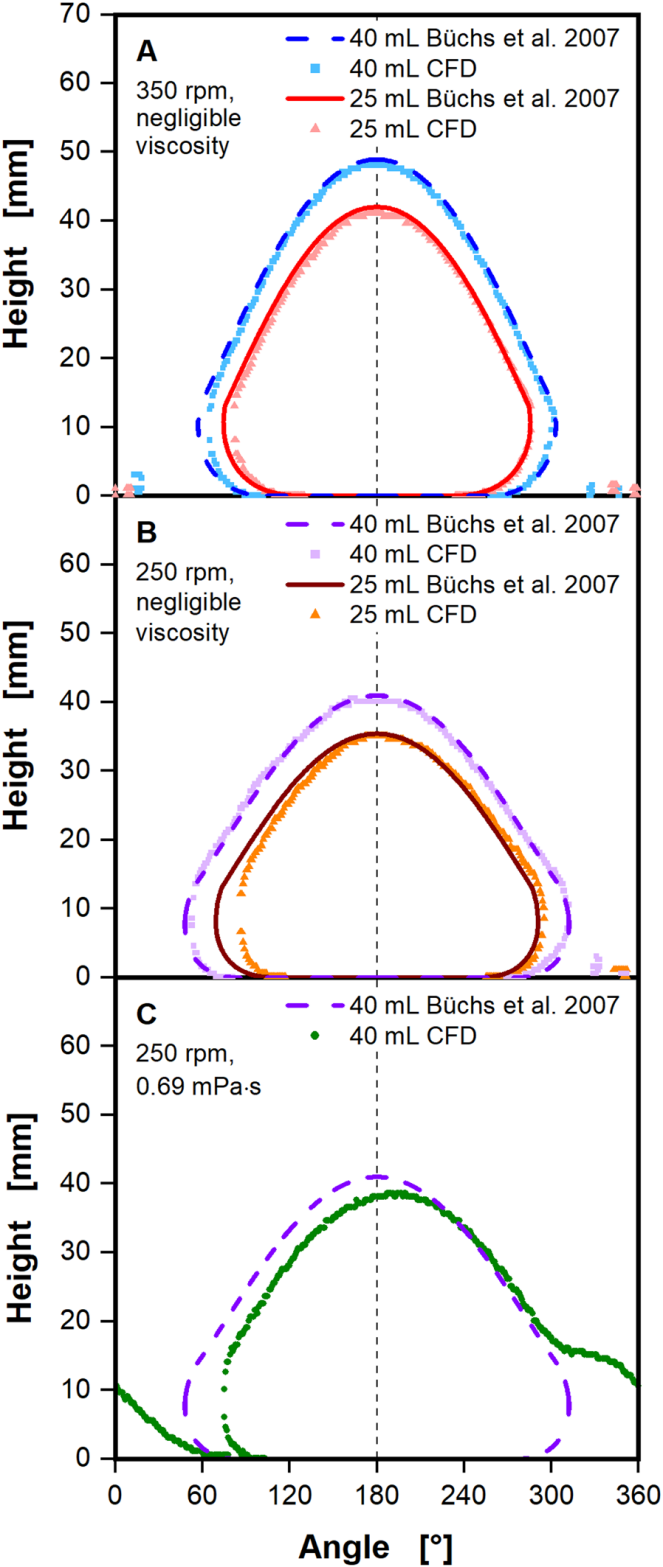
Liquid contact line calculated by the CFD model, compared to the simplified mechanistic model from Büchs et al. assuming negligible viscosity^51^. The height of the liquid contact line is shown in mm, viewed from the center of the shake flask, rotating around the z-axis (see Fig. 1C). For CFD results, the first wall layer, closest to the flask wall is sampled. As indicated by the vertical dashed black line in (**A**), (**B**) and (**C**) the centrifugal force is pointing in the direction of 180°. Calculations of the liquid contact line with the simplified mechanistic model by Büchs et al. entirely neglect viscosity^51^. For comparison, a vanishingly small viscosity was chosen for the CFD model in (**A**) and (**B**). In (**C**) a realistic waterlike viscosity was simulated with the CFD model instead. Simulated conditions: Viscosity (η) = 1·10^–10^ Pa∙s (**A**, **B**) and 0.69 mPa∙s (**C**), shaking diameter (d_0_) = 2.5 cm, filling volume (V_L_) = 25 mL and 40 mL, shaking frequency (n) = 350 rpm (**A**) and 250 rpm (**B**, **C**), surface tension (σ) = 70 mN/m, contact angle (θ) = 20°, temperature (T) = 37 °C (**C**).

In the initial phase of model tuning, the liquid contact lines (Fig. 1, red dashed lines) extracted from the CFD model and simplified mechanistic model^51^, were compared at 25 and 40 mL filling volumes and shaking frequencies of 350 (Fig. 2A) and 250 rpm (Fig. 2B). The liquid contact lines are not depicted in a 3D-representation, as illustrated in Fig. 1, but rather in a 2D-representation by rotating around the central z-axis of the shake flask (Fig. 1C). The height of the contact line over one rotation is depicted. The 3D-representations are not depicted because they quickly become incomprehensible for the comparison between CFD and experiments. An example of a 3D-representation is included in the supplement (see Fig. S1).

Due to the fact that the simplified mechanistic model completely disregards the influence of viscous forces on the liquid distribution, it was necessary to generate comparable conditions in the CFD model. Since entirely removing viscous forces from the CFD model is non-trivial, a viscosity of 1·10^–27^ Pa s was chosen for the comparison instead. It should be noted that the numerical diffusion, due to the discretization of the convective momentum transport, introduces an additional diffusive momentum transport. The overall diffusive momentum transport should, however, remain sufficiently low for a comparison to the simplified mechanistic model. Excluding viscous forces results in a completely symmetric liquid contact line, as can be seen in Fig. 2A and B for the simplified mechanistic model^51^ and the CFD model, respectively. In addition, the bulk liquid and maximal liquid height are perfectly aligned with the direction of the centrifugal force, indicated by the vertical dashed black line in Fig. 2. Given that viscous forces were not entirely eliminated from the CFD simulations, small deviations were anticipated. Yet, liquid contact lines for the CFD remain highly symmetrical, only showing slight deviations in the leading edge (left flank of the contact line) for both filling volumes at 350 rpm and 40 mL at 250 rpm. At a filling volume of 25 mL and a shaking frequency of 250 rpm (Fig. 2B), the largest discrepancy between the CFD and the mechanistic model can be seen. The leading edge extracted from the CFD model is approximately 15° behind the leading edge of the simplified model, resulting in a minor asymmetry in the CFD calculation's contact line. In Fig. 2C the simplified mechanistic model is compared to a CFD simulation with a realistic, waterlike viscosity of 0.69 mPa∙s at the previous shaking parameters of 40 mL and 250 rpm. This example illustrates the effect of viscous forces on the observed liquid contact line. The entire bulk liquid and its maximal liquid height are moved against the shaking motion and positioned about 15° after the direction of the centrifugal force. This shift occurs along the entire leading edge of the liquid contact line, reaching a maximum of nearly 30° at the liquid contact line's widest point. As discussed, this shift of the leading edge of the liquid contact line against the shaking direction has been observed previously^40–44^ and was recorded by Azizan et al. in their liquid distribution data^37,38^. The observed shift with increasing viscosity may also account for the small, yet noticeable differences between the CFD and simplified mechanistic model in Fig. 2A and B. In addition, the effects of viscous forces on the trailing liquid (right flank of the contact line) are visible in the CFD calculation in Fig. 2C. A clear, extended tail from approximately 300° onward, with a visible shoulder at a liquid height of about 15 mm can be seen. In Fig. 2 the shake flask is modelled as a quarter torus for the lower part and a cone for the upper part, resulting in a sharp transition between the lower and upper part of the shake flask at a height of 14.5 mm (refer to Fig. 7A), which is close in height to the extended tail. Consequently, the extended tail after 300° in the CFD calculation is most likely formed due to this sharp transition. The geometric model of the shake flask as a quarter torus was initially chosen by Büchs et al.^51^ for the simplified mechanistic model and replicated in the CFD accordingly.

**Figure 3:**
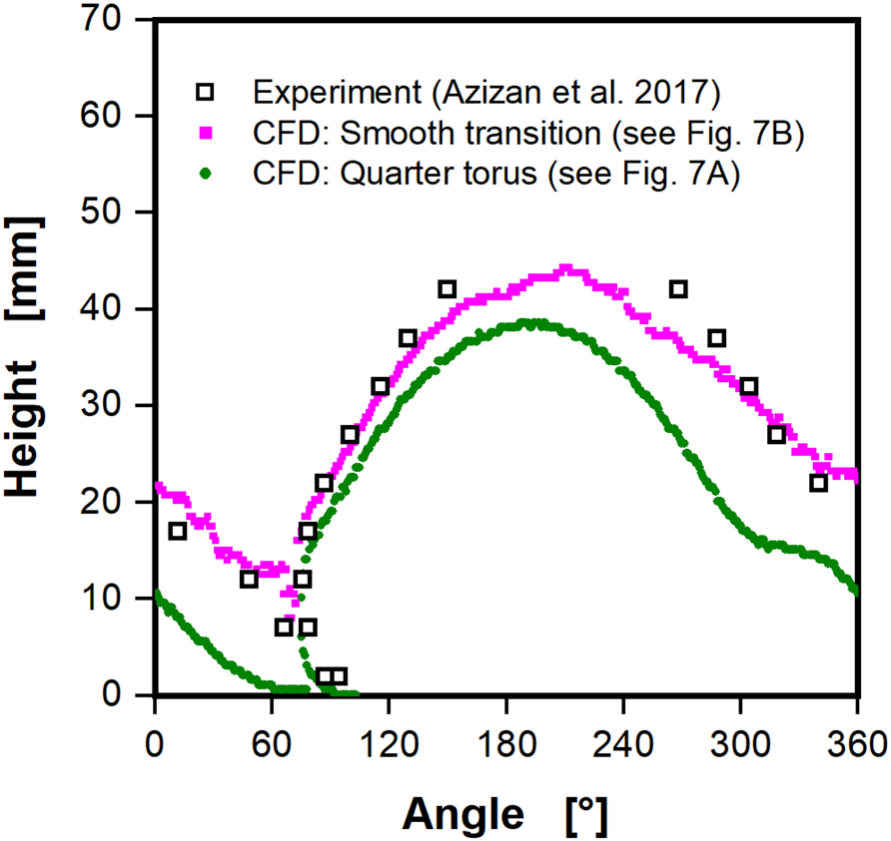
Influence of the assumed shake flask geometry on the liquid contact line. The liquid contact line is shown in mm, viewed from the center of the shake flask, rotating around the z-axis (see Fig. 1C). The underlying shake flasks geometry is either modeled as a quarter torus with an added cone for the upper part or with a smooth transition between torus and cone segment (see Fig. 7A and B, respectively). CFD results are compared with experimental data by Azizan et al.^37,38^. Simulated conditions: Viscosity (η) = 0.69 mPa∙s, shaking diameter (d_0_) = 2.5 cm, filling volume (V_L_) = 40 mL, shaking frequency (n) = 250 rpm, surface tension (σ) = 70 mN/m, contact angle (θ) = 20°, temperature (T) = 37 °C.

Although the simplified mechanistic model verifies the CFD model's accuracy for negligible viscosity, it cannot validate the CFD model for realistic viscosities. Consequently, CFD simulations with viscosities comparable to water and higher must be compared to experimental data. In Fig. 3, the experimental data from Azizan et al.^37,38^ is compared with CFD simulations at waterlike viscosity. In a first attempt, the CFD simulation from Fig. 2C was compared to the experimental data. This simplified approximation of the real shake flask geometry as a quarter torus, however, produced unsatisfactory results. Although, the liquid contact line of the CFD simulation with a quarter torus geometry in Fig. 3 closely matches the experimental data in overall shape and position, the maximal liquid height and tail from 180° onward are underestimated by the CFD model by up to 15 mm. Notably, the shoulder in the tail region at roughly 300° is not observed in the experimental data in Fig. 3. Recall that this shoulder in the tail region appears at the height of the intersection of the quarter torus and cone of the geometric model of the shake flask (see Fig. 7A). Therefore, the sharp transition at the intersection of quarter torus and cone was suspected to be at fault for the poor CFD model performance in the tail region, when compared to the experimental data. Hence, the modelled shake flask geometry was adjusted. The lower torus part was extended past a quarter torus, until it was in line with the slope of the cone part (see Fig. 7B). This approach leads to a smooth transition between the torus and cone and much more closely resembles the geometry of the real shake flask (see Fig. S2). Changing the geometry to a smooth transition, eliminates the issues in the tail region and maximal liquid height to a large extent. The liquid contact line nearly coincides with the experimentally determined liquid contact line and the maximal liquid height of 42 mm. Solely, a minor difference in the tail of the liquid contact line can be seen. Notably, a plateau in the tail region of the CFD simulations is no longer present. In conclusion, the smooth transition replicates the real shake flask geometry (see Fig. S2) significantly better and was crucial to the satisfactory performance of the CFD model. In light of this, the geometry with a smooth transition is used in all subsequent CFD simulations.

**Figure 4:**
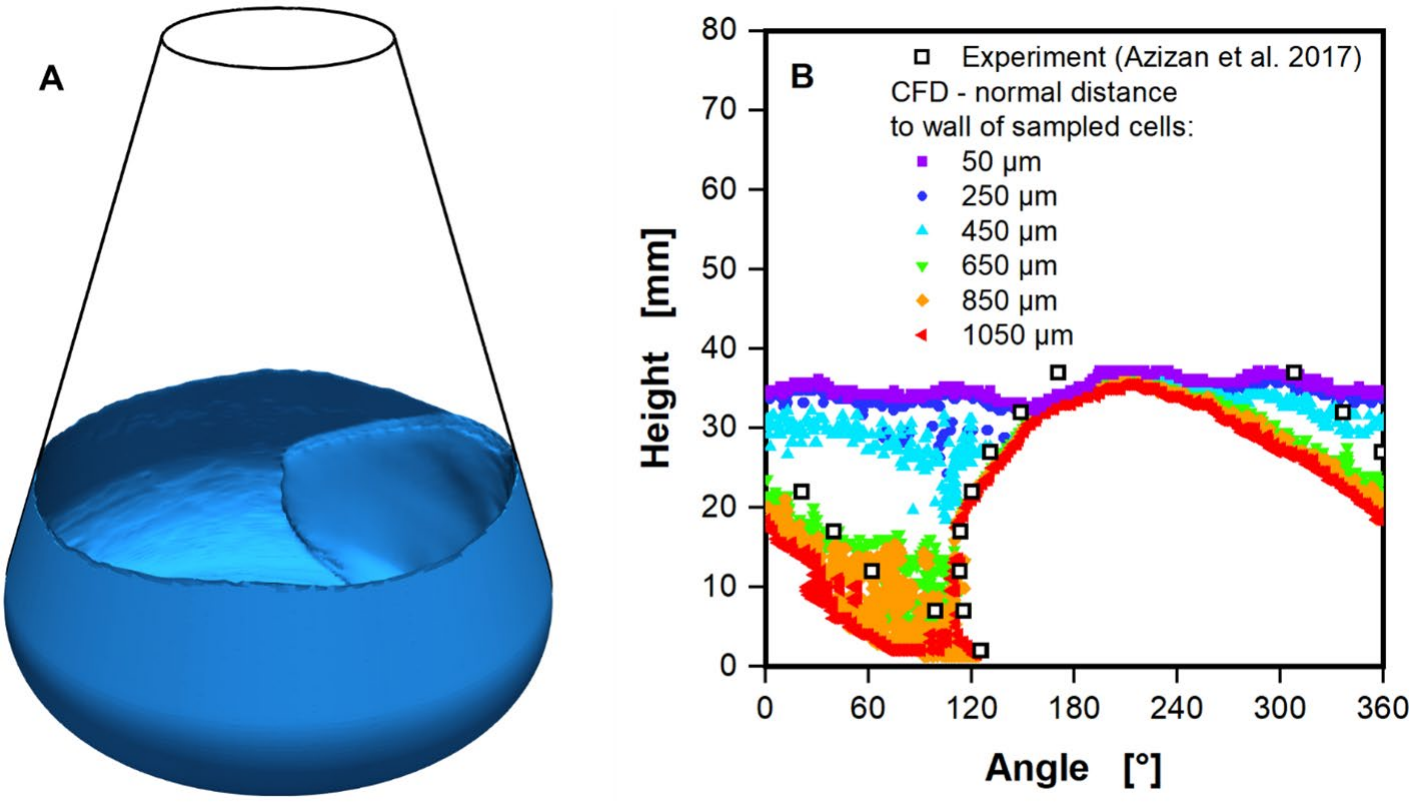
Impact of the liquid film on the extraction of the liquid contact line from CFD calculations at elevated viscosity. (**A**) 3D image of the calculated CFD case, showing a liquid film at the inner flask wall along the entire circumference of the shake flask, up to the maximal liquid height. (**B**) Liquid contact lines from CFD calculations, extracted by numerically sampling at multiple distances normal to the shake flask wall (as indicated in Fig. 8A), aiming to exclude the resolved liquid film, are shown. Results for the liquid contact line from CFD are compared to experimental data by Azizan et al.^37,38^. Simulated conditions: Viscosity (η) = 16.7 mPa∙s, shaking diameter (d_0_) = 2.5 cm, filling volume (V_L_) = 40 mL, shaking frequency (n) = 250 rpm, surface tension (σ) = 70 mN/m, contact angle (θ) = 20°, temperature (T) = 25 °C.

Following the selection of a shake flask geometry, the effects of an increase in viscosity on CFD model performance was investigated. Consequently, Fig. 4 depicts the results of a CFD simulation at an elevated viscosity of 16.7 mPa∙s. Increasing the viscosity from waterlike viscosity to 16.7 mPa∙s results in a reduction of the Reynolds number from roughly 31.000 to roughly 1.600. Figure 4A shows the three-dimensional liquid distribution computed by the CFD model. In addition to the leading edge of the bulk liquid, a liquid film on the inner flask wall over the entire circumference up to the maximal liquid height is visible. Hence, the liquid contact line of the CFD simulation cannot be extracted directly at the flask wall (normal distance of 50 µm), as done for Figs. 2 and 3. Instead, the liquid contact line is extracted multiple times, increasing the normal distance between sampled cells and the shake flask wall with each extraction (compare Fig. 8A). Figure 4B depicts liquid contact lines at these normal distances. For small normal distances between 50 and 250 µm, almost constant liquid heights between 35 and 37 mm are extracted, representing the liquid film over the entire flask circumference. In contrast, the large normal distances of 650, 850 and 1050 µm surpass the liquid film and, therefore, represent the liquid contact line of the bulk liquid. Liquid contact lines at the high normal distances align well with the experimental data in the leading edge and overall position of the bulk liquid. Nonetheless, variations in the contact line can be observed in the tail region. These disparities, however, are not surprising. The leading edge is characterized by a stark difference in the liquid thickness normal to the wall, as evident in Fig. 4A. In contrast, it is challenging to define the limits of the tail region, due to the seamless transition between the tail region of the bulk liquid and the liquid film (compare Fig. S3B). Consequently, precisely defining the tail region is somewhat arbitrary and much more error-prone, compared to the detection of the leading edge. This is the simple, physical reality of the flow in shake flasks and affects the detection of the tail region in experiments and CFD alike.

Concerning the observed liquid films in the CFD simulations, it should be noted that the formation of such liquid films in shake flask experiments and its importance for the gas–liquid mass transfer has already been discussed in the literature^52–55^. Such liquid films can also be seen in photographs of shake flask experiments in the supplementary data (Figs. S3B, S4B and S5B). Hermann conducted approximate measurements of the liquid film thickness in shake flasks (see Figs. S6, S7 and S8)^56^. He used a measurement apparatus comparable to the one from Azizan et al. for the liquid contact lines^37^, but with a single sensor at a height of 20.6 mm. Hermann assessed liquid film thicknesses of 50 and 800 µm at 1 and 35 mPa∙s, respectively (see Fig. S8). Comparatively, the extraction of liquid contact lines at numerous distances normal to the flask wall in the CFD model reveals the liquid film thickness to be between 450 and 650 µm (Fig. 4B), being slightly thinner near the maximal liquid height due to gravity. Taking into account the lower viscosity of 16.7 mPa∙s in the CFD model, the results are consistent. In CFD simulations with a viscosity comparable to water, however, no liquid film is observed, as shown in Fig. 3. This is most likely caused by an insufficient mesh resolution for liquid films with an expected thickness of about 50 µm. The thinnest wall layer of mesh cells with an approximate edge length of 140 µm perpendicular to the wall (see Fig. 8A) are likely too large to resolve these thin liquid films. Resolving the thin liquid film for waterlike viscosity might be possible with a finer mesh, an adaptively refined mesh, where mesh cells are refined during CFD computation or with a Direct Numerical Simulation. All these approaches, however, lead to significantly longer computation times, with extreme computation times in the case of Direct Numerical Simulations. The comparison between the CFD results and the experimental liquid contact lines from Azizan et al.^37,38^ does, generally, not require consideration of the liquid film. Therefore, the faster computation time of the fixed meshing is preferable and was chosen for all simulations in this work. Additionally, the liquid film contributes very little to the overall volumetric power input, as can be seen later on, when volumetric power inputs form model and correlation are compared.

Due to the possibility of small underestimations of the maximal liquid height in the CFD simulations in Figs. 3 and 4B, some of the material parameters (taken from VDI Heat Atlas^57^) were selected for a parameter study prior to simulating a larger data set of shaking conditions. Surface tension, contact angle and the slope of the upper cone part of the shake flask geometry were selected, as variables that could potentially affect the maximal liquid height. Adjustment of the parameters were small and on a realistic scale, and unidirectional, favoring potentially greater maximum liquid heights. Thus, a 10% decrease in surface tension, a 15° decrease in contact angle, and a 1° decrease in the slope of the cone were simulated. Resulting liquid contact lines are shown in the supplementary data in Figs. S9, S10 and S11. No appreciable differences in the overall liquid contact line and maximal liquid height were observed for any of the three adjustments. Consequently, the standard parameters from the previous simulations were used for a larger set of CFD simulations.

**Figure 5:**
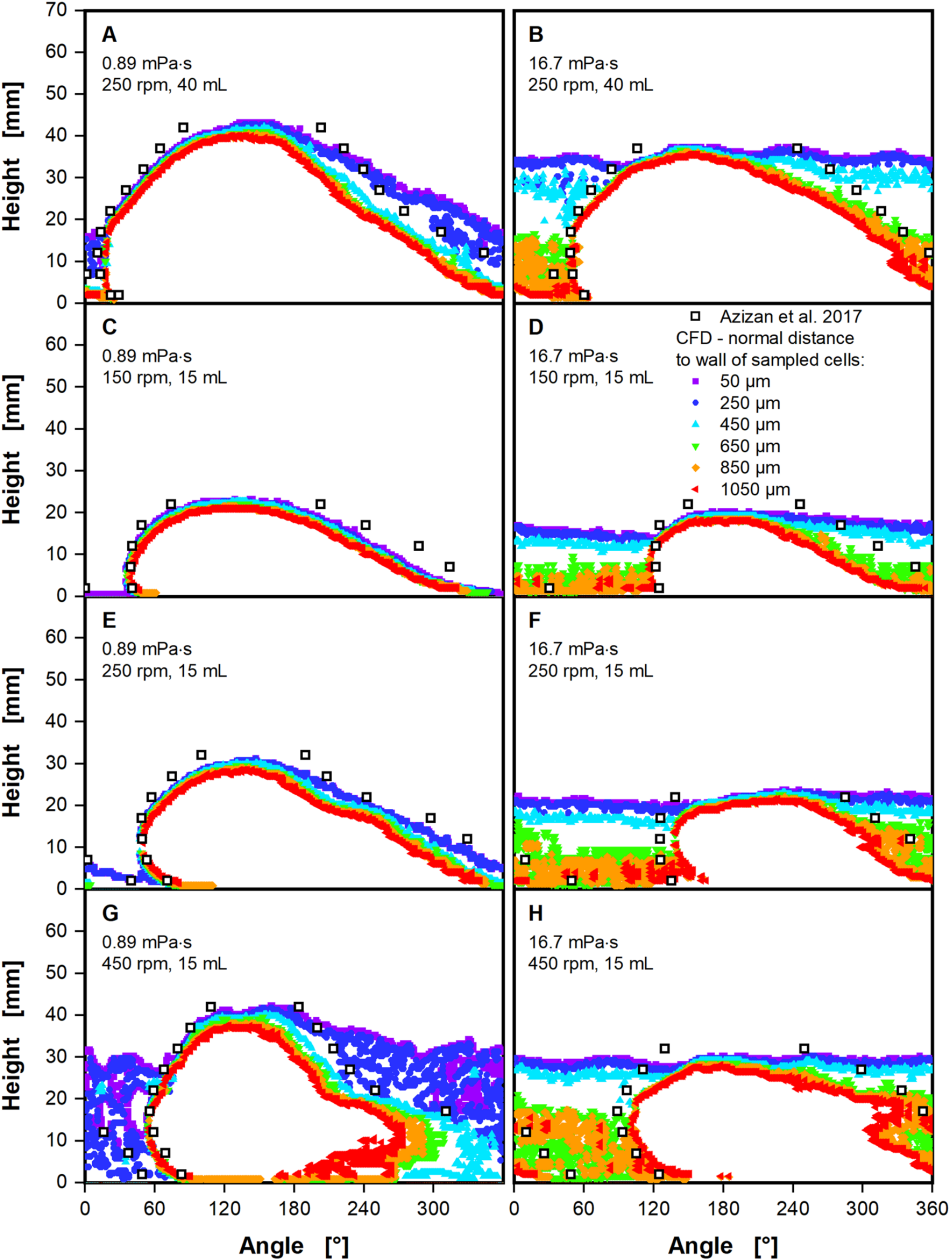
Liquid contact lines calculated by the CFD model, compared to experimental data by Azizan et al.^37,38^ for a variety of shaking conditions. The liquid contact lines are shown in mm, viewed from the center of the shake flask, rotating around the z-axis (see Fig. 1C). Liquid contact lines from CFD were extracted at multiple distances from 50 to 1050 µm normal to the shake flask wall (as indicated in Fig. 8A) to exclude the liquid film (see Fig. 4A). Simulated conditions: Shaking diameter (d_0_) = 2.5 cm, surface tension (σ) = 70 mN/m, contact angle (θ) = 20°, temperature (T) = 25 °C. (**A**, **C**, **E**, **G**) Cases with a low viscosity of 0.89 mPa∙s. (**B**, **D**, **F**, **H**) Cases with a higher viscosity of 16.7 mPa∙s. (**A **+ **B**) filling volume (V_L_) = 40 mL, shaking frequency (n) = 250 rpm. (**C** + **D**) filling volume (V_L_) = 15 mL, shaking frequency (n) = 250 rpm. (**E** + **F**) filling volume (V_L_) = 15 mL, shaking frequency (n) = 150 rpm. (**G** + **H**) filling volume (V_L_) = 15 mL, shaking frequency (n) = 450 rpm.

In order to thoroughly validate the model against the experimental data provided by Azizan et al.^37,38^, a larger set of CFD simulations were conducted following the optimization of the model. For the larger set of CFD simulations, nine combinations of shaking conditions were selected, including shaking frequencies between 150 and 450 rpm and filling volumes between 15 and 40 mL. For each of the nine combinations, experimental data on the liquid distribution by Azizan et al.^37,38^ is available. CFD simulations were performed for a waterlike viscosity of 0.89 mPa∙s and an elevated viscosity of 16.7 mPa∙s. A subset of four of the shaking conditions for both viscosities are shown in Fig. 5. Figure 5A, C, E and G depict the results at the waterlike viscosity, while Fig. 5B, D, F and H depict the results at the moderate viscosity of 16.7 mPa∙s. The extraction of liquid contact lines at multiple normal distances described for Fig. 4 has been performed not only for the moderate viscosity, but also for waterlike viscosity. CFD simulations for Fig. 5A and B have already been discussed and are depicted in Figs. 3 and 4. The remaining subfigures depict the results for a filling volume of 15 mL at shaking frequencies of 150 rpm (Fig. 5C and D), 250 rpm (Fig. 5E and F) and 450 rpm (Fig. 5G and H). CFD model performance at these shaking conditions is comparable to the ones already discussed. Overall shape and position of the leading edge align nicely between experiment and CFD simulation, as long as liquid films are excluded by analyzing the larger normal distances to the shake flask wall. Only for shaking frequencies of 250 and 450 rpm at a moderate viscosity of 16.7 mPa∙s, as depicted in Fig. 5F and H, are there notable deviations in the liquid contact line between experimental data and CFD model. In both cases, the shape of the leading edge appears to be the same, yet, occurs approximately 15° later in the CFD model than the experimental data suggests. Notably, the CFD model accurately predicts the shift of the liquid against the shaking direction as viscosity increases. In some of the simulated conditions, the extracted maximum liquid heights may be slightly lower than the experimentally determined values. This affects waterlike viscosity (Fig. 5E) and moderate viscosity (Fig. 5D and H) alike, showing differences of up to 3 mm between the two. Taking into consideration the already discussed issues of defining the tail region in the context of Fig. 4B, the tail region is suitably matched by the CFD simulations. This holds true especially for cases with a waterlike viscosity.

Curiously, the tail region no longer exhibits a consistent slope at high shaking frequencies. At 450 rpm at waterlike viscosity (Fig. 5G) the liquid height remains almost constant at 20 mm from 200° onwards, only declining slightly. Figure 5H depicts the formation of a spike-like feature at 450 rpm for the moderate viscosity at a similar height and position in the tail region. As previously discussed in the context of Fig. 4, the extraction of liquid contact lines at multiple distances from the flask wall in the CFD model can be used to estimate the thickness of formed liquid films. Contrary to previous findings, a partially formed liquid film with a thickness of approximately 250 µm can be observed in CFD cases with waterlike viscosity, and at the highest shaking frequency of 450 rpm (Fig. 5G). For the moderate viscosity of 16.7 mPa∙s, a film thickness ≥ 500 µm is found in the CFD simulations. As previously mentioned, Hermann provides approximations for the liquid film thickness in shake flasks^56^. For viscosities of 1 and 35 mPa∙s, a liquid film thickness of 50 and roughly 800 µm, respectively, were found, confirming the CFD model results.

### Calculation of volumetric power inputs from CFD simulations

**Figure 6:**
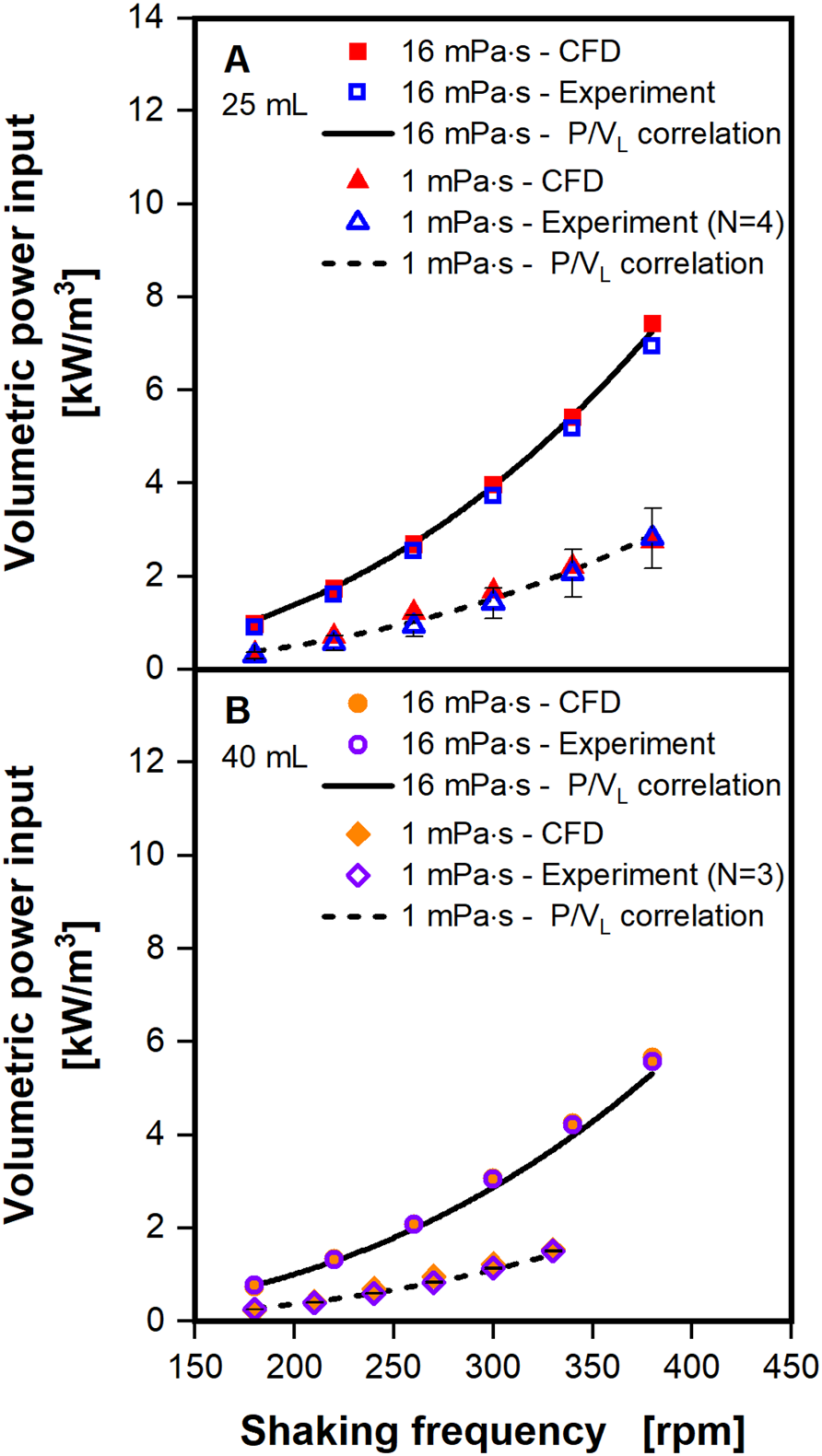
Comparison of volumetric power input obtained from CFD calculations with experimental data and the correlation of Büchs et al.^44^. The volumetric power inputs calculated from CFD utilizing the energy dissipation rate (see Eq. 23) are shown (filled symbols). Results are compared to experimental results (open symbols) and values calculated with the volumetric power input correlation (line and dashed line) from Büchs et al.^44^. Replicates of the experimentally determined volumetric power inputs and corresponding error bars exist only for the low viscosity of 1 mPa∙s at 25 (**A**) and 40 (**B**) mL. Simulated conditions: Viscosity (η) = 1 mPa∙s and 16 mPa∙s, shaking diameter (d_0_) = 2.5 cm, filling volume (V_L_) = 25 mL (**A**) and 40 mL (**B**), shaking frequency (n) = 180–380 rpm, surface tension (σ) = 70 mN/m, contact angle (θ) = 20°, temperature (T) = 20 °C.

The volumetric power input was chosen as the first benchmark to be calculated from the CFD model, demonstrating the usefulness of a well validated CFD model. CFD simulations were run at filling volumes of 25 and 40 mL, viscosities of 1 and 16 mPa∙s, shaking frequencies from 180 to 380 rpm and a shaking diameter of 25 mm, calculating the volumetric power input based on the energy dissipation (Eqs. 20 and 23). Values calculated from the CFD model are compared to experimental data and values calculated based on the Ne^′^-Re-correlation by Büchs et al.^43,44^ (Fig. 6). Triplicates for the experimental data at a viscosity of 1 mPa∙s are available at both filling volumes, but not for the higher viscosity of 16 mPa∙s, as indicated by the error bars. When comparing only experimental data and correlation values, both from Büchs et al.^43,44^, a remarkably small deviation can be seen. It should be noted that in the Ne´ and, therefore, in the Ne´-Re-correlation the shaking diameter was omitted for the sake of simplicity^43,44^. An increase in the shaking diameter, however, leads to an increase in the centrifugal force and greater liquid height and, therefore, an increase in volumetric power input. This increase in the volumetric power input is visible in the experimentally determined volumetric power inputs from Büchs et al.^43,44^. Extending the shaking diameter from 2.5 to 5 cm increased the volumetric power input by roughly 13% for waterlike and moderate viscosity. This effect is illustrated by a parity plot of the original experimentally determined volumetric power inputs from Büchs et al., which is included in the supplement (see Fig. S12). In addition, the effect of the shaking diameter on volumetric power inputs was also observed in our CFD simulations of various shaking diameters (data will be included in a future publication). The low deviation between the experimental data and the correlation in Fig. 6 is, therefore, somewhat unexpected.

Volumetric power inputs, computed based on the proposed CFD model, also align exceptionally well with the experimental data and correlation. CFD, experiment, and correlation differ by a maximum of 0.5 kW/m^3^ across all simulated conditions, with an average deviation of 0.01 kW/m^3^. As anticipated, the volumetric power input increases as shaking frequency and viscosity increase, and when filling volume decreases. Note that these trends must only hold true if the out-of-phase phenomenon is avoided^38,44^.

As discussed in the context of Figs. 4 and 5 the liquid film is not completely resolved by the CFD model for waterlike viscosity, except for the highest shaking frequency of 450 rpm. Nevertheless, the calculated volumetric power inputs are correct, suggesting that the liquid film contributes negligible power to the liquid. This finding is expected, as the energy dissipation is based on the velocity gradients, as it is utilized in the CFD model (Eq. 23). In the liquid film, movement is mainly caused by the gravitational force, causing small velocity gradients. Comparably massive velocity gradients are observed in the rotating bulk liquid as it rolls over the shake flask wall. Hence, the bulk liquid is the major contributor to the volumetric power input in shake flask experiments.

### Conclusions

An OpenFOAM CFD model for shake flasks was established and its liquid contact line compared to the simplified mechanistic model by Büchs et al.^51^, showing good agreement when assuming negligible viscosity. When viscosity is included, the importance of correctly modelling the shake flask geometry was highlighted. In this study, a geometry composed of an upper cone and a lower partial torus was employed. Crucially, the partial torus was extended until its curvature was in line with the cone, resulting in a smooth transition between the two. When the viscosity was increased from waterlike to a moderate viscosity of 16.7 mPa∙s, a liquid film of approximately 500 µm was observed, comparable to the 800 µm experimentally determined by Hermann for 35 mPa∙s^56^. By extracting the liquid contact lines at multiple distances, normal to the shake flask wall, this liquid film could be disregarded and the bulk liquid compared to experimental data. The CFD model demonstrated excellent agreement with the experimental data provided by Azizan et al.^37,38^ in terms of overall liquid position, relative to the centrifugal force, and overall shape of the contact line. This holds true across all tested shaking parameters, including shaking frequencies ranging from 150 to 450 rpm and filling volumes ranging from 15 to 40 mL, at waterlike and moderate viscosity. Furthermore, the established CFD model was used to compute the volumetric power inputs, based on the energy dissipation. The resulting volumetric power inputs deviated on average by 0.01 kW/m^3^ from the experimental data of Büchs et al. and their correlation^43,44^. These excellent agreements signify the importance of the comprehensive validation. In the future the CFD model can be extended to calculate mixing times, shear forces and the gas–liquid mass transfer. As long as the liquid distribution and, therefore, convection and energy dissipation are correctly modelled, the mixing times and shear forces should already be implicitly solved by our model.

In summary, the established CFD model was validated by liquid distributions and could already be used to calculate volumetric power inputs. The model will be used to extent and improve common correlations for the shake flasks, like volumetric power input, while alleviating the need for additional experiments to do so. Furthermore, it is readily applicable to novel shaken bioreactor designs of comparable scale for which no experimental data or correlations for important engineering parameters such as power inputs exist. The presented CFD model has, in fact, already been used to aid in the design of a specific shaken bioreactors with concentric rings (termed “perforated ring flask”) by Hansen et al.^23^. Additionally, CFD models for shake flask will proof valuable in the fundamental understanding of shaken bioreactors and in the scale-up of bioprocesses.

## Materials and methods

### The interFoam solver

The CFD simulations in this work were performed with OpenFOAM version 9, released by the OpenFOAM Foundation^58^. For an accurate estimation of the liquid movement in shake flasks, the interface between gas and liquid phase must be resolved. When handling incompressible and immiscible fluids, the Volume of Fluid (VOF) method is the standard approach for this situation. Instead of solving a set of Euler equations for each fluid separately, the VOF method needs to solve only one set of Euler equations for the average conditions in each mesh cell. The VOF method only requires a single extra conservation equation for the volume fraction. This leads to a significantly lower computation effort, compared to “true” two-phase simulation approaches. In the simulations in this work the interFoam solver was used for performing the VOF simulations. It solves the standard conservation equations for mass and momentum for incompressible fluids:

1 $$\nabla \mathbf{U}=0$$

2 $$\frac{\partial }{\partial t}\rho\cdot \mathbf{U}+\nabla \left(\rho\cdot \mathbf{U}\cdot \mathbf{U}\right)=-\nabla p+\nabla (\tau +{\tau }_{t})+\rho\cdot {\varvec{g}}+{\mathbf{F}}_{\sigma }$$

where **U**, ρ, p, τ, τ_t_, **g** and **F**_σ_ are the velocity in m/s, density in kg/m^3^, pressure in Pa, viscous and turbulent stress in Pa, gravity acceleration on the reverse z-axis in m/s^2^ and surface tension in N/m^2^. Additionally, the VOF method implements a conservation equation for the interface:

3 $$\frac{\partial }{\partial t}{\alpha }_{i}+\left(\mathbf{U}\cdot \nabla {\alpha }_{i}\right)=0$$

where α_i_ encodes the volume fraction of phase i. Accordingly, the following constraint on α_i_ exists:

4 $$\sum {\alpha }_{i}=1$$

In this work only two phases are simulated, a liquid and a gas phase i.e., water and air. If the volume fraction for the liquid phase α_L_ = 1, the cell in question is entirely filled with water and for α_L_ = 0 with air. In the case of 0 < α_L_ < 1 the cell is part of the free surface between both phases. Furthermore, the volume fraction is used to determine the average density and viscosity as follows:

5 $$\rho ={\alpha }_{L}\cdot {\rho }_{L}+\left(1-{\alpha }_{L}\right)\cdot {\rho }_{G}$$

6 $$\eta ={\alpha }_{L}\cdot {\eta }_{L}+\left(1-{\alpha }_{L}\right)\cdot {\eta }_{G}$$

where ρ_L_, ρ_G_, $\eta$_L_ and $\eta$_G_ are the density and viscosity of the liquid and gas phase, respectively. The surface tension is modelled as a continuum surface force (CSF)^59,60^, calculated as follows:

7 $${\mathbf{F}}_{\sigma }=\sigma\cdot \kappa\cdot \nabla \alpha$$

8 $$\kappa =\nabla \left(\frac{\nabla \alpha }{\left|\nabla \alpha \right|}\right)$$

where σ is the surface tension constant in N/m^2^ and κ is an approximation of the curvature at the interface^59,60^. Previously, a critical Reynolds number for turbulent flow of 60,000 in shake flasks has been proposed by Peter et al.^61^. Although a Reynolds number of 60,000 is not achieved under usual shaking conditions for 250 mL shake flasks, some experiments in this work come close (Re ~ 55,000 for conditions in Fig. 5G). Hence, at least transitional flow is to be expected for some of the simulated conditions. Nevertheless, a simulation for fully laminar conditions was conducted in preliminary calculations. Volumetric power input for this fully laminar simulation was, however, only half of the experimentally determined volumetric power input. Therefore, the k-omega Shear Stress Transport (k-omega SST) turbulence model was utilized in the simulations^62^. The k-omega SST model implements the k-epsilon model in the free shear stream, but switches to the k-omega model for better model performance up to the shake flask wall, through the viscous sublayers. This makes the k-omega SST model applicable as a low Reynolds number turbulence model.

In OpenFOAM the k-omega SST model from Menter et al.^62^ is implemented. They give the conservation equations for the turbulent kinetic energy (*k*) and the specific energy dissipation rate (ω) as follows:

9 $$\rho \frac{\partial k}{{\partial t}} + \rho \frac{{\partial \left( {U_{i} k} \right)}}{{\partial x_{i} }} = \tilde{P} - \underbrace {{\beta^{*} \rho k\omega }}_{\begin{subarray}{l} dissipation \\ \;\;\;\;\;of\;k \end{subarray} } + \frac{\partial }{{\partial x_{i} }}\left[ {\left( {\eta + \sigma_{k} \eta_{t} } \right)\frac{\partial k}{{\partial x_{i} }}} \right]$$

10 $$\rho \frac{\partial \omega }{\partial t}+\rho \frac{\partial \left({U}_{i}\omega \right)}{{\partial x}_{i}}=\frac{\gamma \rho }{{\eta }_{t}}\widetilde{P}-\beta \rho {\omega }^{2}+\frac{\partial }{{\partial x}_{i}}\left[\left(\eta +{\sigma }_{\omega }{\eta }_{t}\right)\frac{\partial \omega }{{\partial x}_{i}}\right]+2(1-{F}_{1})\rho {\sigma }_{\omega 2}\frac{1}{\omega }\frac{\partial k}{{\partial x}_{i}}\frac{\partial \omega }{{\partial x}_{i}}$$

with $\widetilde{P}$ being the limiter of the production of turbulent kinetic energy, η, η_t_ the viscosity and turbulent viscosity, respectively and F_1_ a blending function. β^*^, σ_k_, γ and σ_w_ are model coefficients. The production limiter is given as:

11 $$\widetilde{P}={\text{min}}\left({\eta }_{t}\frac{\partial {U}_{i}}{{\partial x}_{j}}\left(\frac{\partial {U}_{i}}{{\partial x}_{j}}+\frac{\partial {U}_{j}}{{\partial x}_{i}}\right),10 {\beta }^{*}\rho k\omega \right)$$

The blending function F_1_ leads to the transition from the k-omega SST to the k-epsilon model and is defined as:

12 $${F}_{1}={\text{tanh}}\left\{\left\{{\text{min}}\left[{\text{max}}\left(\frac{\sqrt{k}}{{\beta }^{*}\omega y},\frac{500\eta }{{y}^{2}\omega \rho }\right),\frac{{4\rho \sigma }_{w2}}{{CD}_{k\omega }{y}^{2}}\right]\right\}\right\}$$

where y is the normal distance from the nearest wall and CD_kω_ is given as:

13 $${CD}_{k\omega }={\text{max}}\left({2\rho \sigma }_{\omega 2}\frac{1}{\omega }\frac{\partial k}{{\partial x}_{i}}\frac{\partial \omega }{{\partial x}_{i}},{10}^{-10}\right)$$

For the turbulent viscosity, Menter et al.^62^ give the following expression:

14 $$\frac{{\eta }_{t}}{\rho }=\frac{{a}_{1}k}{{\text{max}}({a}_{1}\omega ;|{\text{\bf{S}}}|{F}_{2})}$$

where a_1_ is another model coefficient, |**S**| a scalar measure for the strain rate tensor and F_2_ a second blending function, which is defined as:

15 $${F}_{2}={\text{tanh}}\left[{\left[{\text{max}}\left(\frac{2\sqrt{k}}{{\beta }^{*}\omega y},\frac{500\eta }{{y}^{2}\omega \rho }\right)\right]}^{2}\right]$$

The scalar measure for the strain rate tensor is defined as follows:

16 $$\left|{\text{\bf{S}}}\right|=2\cdot \left[{\left(\frac{\partial u}{\partial x}\right)}^{2}+{\left(\frac{\partial v}{\partial y}\right)}^{2}+{\left(\frac{\partial w}{\partial z}\right)}^{2}\right] +{\left(\frac{\partial u}{\partial y}+\frac{\partial v}{\partial x}\right)}^{2}+{\left(\frac{\partial v}{\partial z}+\frac{\partial w}{\partial y}\right)}^{2}+{\left(\frac{\partial u}{\partial z}+\frac{\partial w}{\partial x}\right)}^{2}$$

where u, v and w are the velocity components in direction of the cartesian coordinates x, y and z. All model coefficients, used in Eqs. (9–16), are given in Table 1. Model coefficients, which are given with subscripts 1 and 2 are also blended with blending function F_1_, according to the following expression:

**Table 1:** Model coefficients of the k-omega SST model.

Model coefficient	Value (-)
${\beta }^{*}$	0.09
${a}_{1}$	0.31
${\sigma }_{k1}$	0.85
${\sigma }_{k2}$	1
${\sigma }_{\omega 1}$	0.5
${\sigma }_{\omega 2}$	0.856
${\beta }_{1}$	0.075
${\beta }_{2}$	0.0828
${\gamma }_{1}$	5/9
${\gamma }_{2}$	0.44

17 $$\varphi ={\varphi }_{1}{F}_{1}+{\varphi }_{2}(1-{F}_{1})$$

Simulations were transient and performed with an adjustable time step, limited by a maximal Courant number of 1 and additionally, a maximal time step of 0.005 s.

Material parameters for the simulations (see Table S1) were taken from the VDI Heat Atlas^57^.

### Modelling the shaking motion

Shake flasks are usually agitated on orbital shakers with a given shaking diameter and shaking frequency. Directly simulating this shaking motion requires a recalculation of the positioning of mesh at each timepoint. To avoid the computational expense of mesh recalculation, the modelling approach from Li et al. was implemented^20^. They reduced the shaking motion down to a cyclic centrifugal force, sweeping around the stationary shake flask. The forces in the x and y direction can be calculated as follows:

18 $${F}_{x}={\overrightarrow{\omega }}^{2}\cdot \frac{{d}_{0}}{2}\cdot {\text{cos}}(\omega\cdot t)$$

19 $${F}_{y}={\overrightarrow{\omega }}^{2}\cdot \frac{{d}_{0}}{2}\cdot {\text{sin}}(\omega\cdot t)$$

where $\overrightarrow{\omega }$, d_0_ and t are the angular velocity in rad/s, shaking diameter in m and point in time in s. All simulations in this work were performed with a runtime of 10 s. In the first 0.5 s of this runtime the shaking frequency linearly ramps up from a standstill to the desired shaking frequency.

### Shake flask geometry and mesh generation

**Figure 7:**
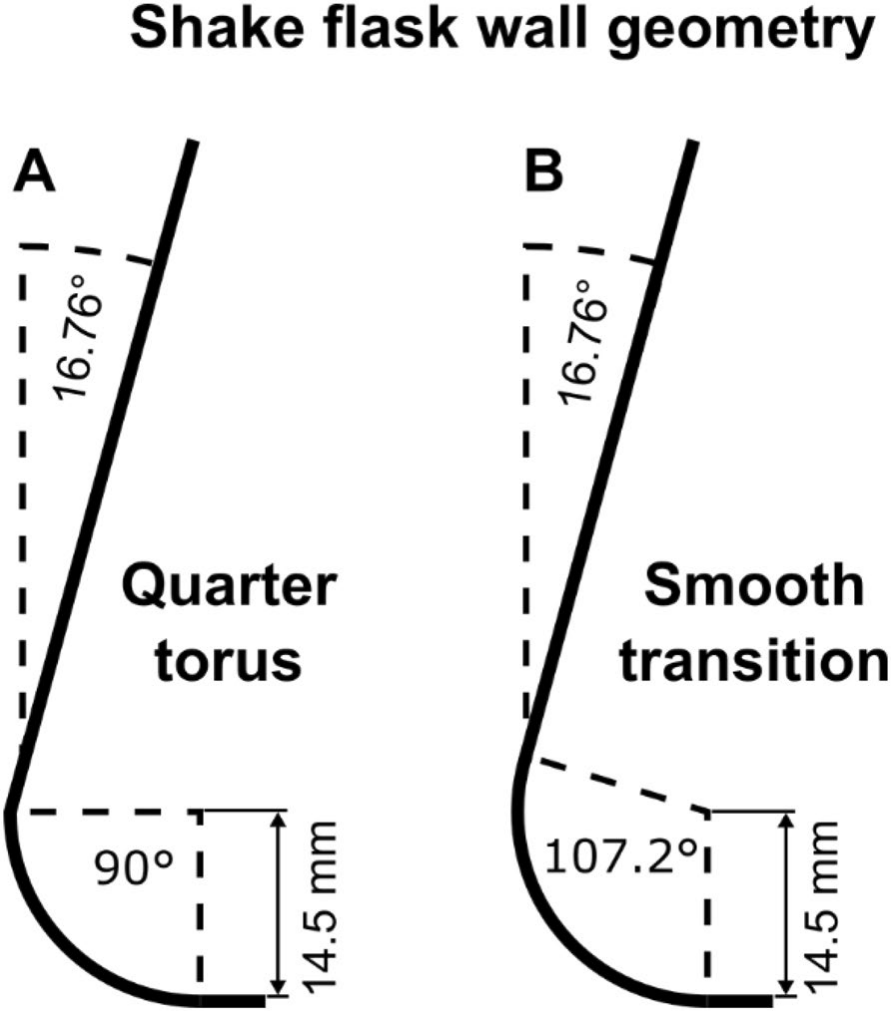
Schematic illustration of the shake flask geometry. Two approaches in modelling the basic shape of shake flasks are shown. (**A**) The shake flask is modelled as a quarter torus (r = 14.5 mm) with an added cone (16.76°) for the upper part, leading to a sharp transition between the segments^51^. (**B**) The curvature of the torus (r = 14.5 mm) is extended until it is in line with the slope of the cone (16.76°), leading to a smooth transition.

**Figure 8:**
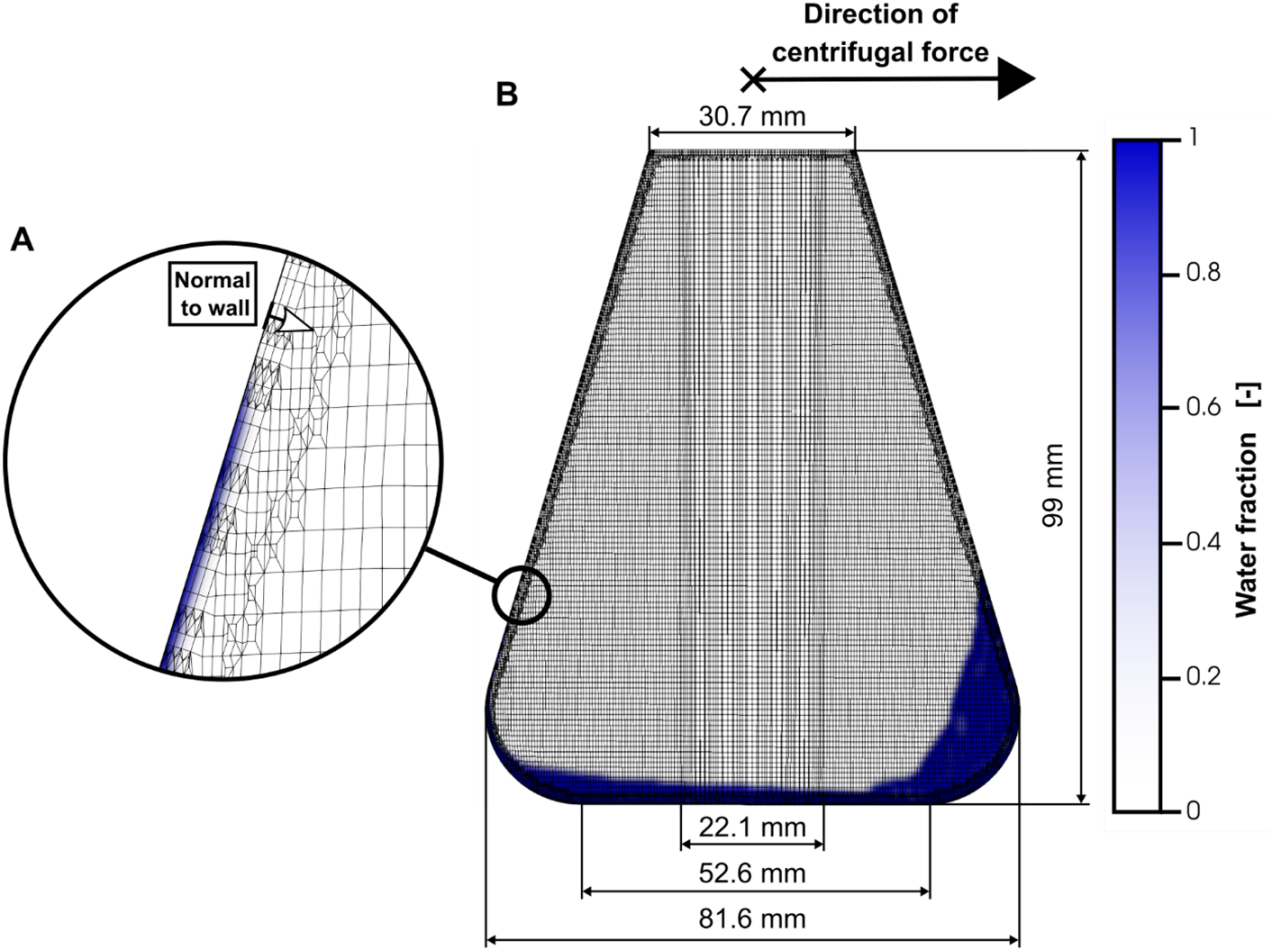
Cross-section of the meshed shake flask. In (**B**) the cross-section of the entire shake flask mesh can be seen. A close-up view of the wall is depicted in (**A**). The 3D model of the shake flask is meshed with the OpenFOAM utility *snappyHexMesh*. First, a cylindrical base mesh is created with *blockMesh*. Two refinement steps (factor 8 increase in mesh resolution), introduced during the castellated meshing of *snappyHexMesh* can be seen. One refinement step is introduced at a diameter of 22.1 mm. A second one can be seen near the shake flask wall in (**A**). Additionally, three wall layers, depicted in (**A**) are introduced. Wall layers are orientated parallel to the shake flask wall, as indicated by the arrow normal to the wall in (**A**). A completely flat bottom with a diameter of 52.6 mm is modelled. In total, the meshed shake flask model consists of roughly 1.4 million cells. The color scale from white to blue indicates the water fraction of the simulated CFD case. Simulated conditions: Viscosity (η) = 16.7 mPa∙s, shaking diameter (d_0_) = 2.5 cm, filling volume (V_L_) = 40 mL, shaking frequency (n) = 250 rpm, surface tension (σ) = 70 mN/m, contact angle (θ) = 20°, temperature (T) = 25 °C.

Generally, the geometry of shake flasks consists of a partial torus for the lower part and an added cone for the upper part of the shake flask. Two approaches to model the intersection between lower torus and upper cone are used in this work (Fig. 7). The first approach is consistent with one in the simplified mechanistic model by Büchs et al.^51^ and is used for the comparison to the simplified mechanistic model. It approximates the lower part as a quarter torus (90°) with an added cone as the upper part of the flask^51^, as illustrated in Fig. 7A, leading to a sharp transition between lower and upper part of the shake flask. In the second approach the curvature of the torus is extended until it is in line with the slope of the added cone (107.2°), as shown in Fig. 7B, creating a smooth transition between lower and upper part. This second approach resembles the real shake flask geometry much more closely (see Fig. S2). All dimensions are otherwise consistent between both approaches. They include a torus radius of 14.5 mm (Fig. 7), maximal diameter of 81.6 mm (Fig. 8B) at a height of 14.5 mm, tapering down to a diameter of 30.7 mm at a height of 99 mm (Fig. 8B), equating to an angle of 16.76° for the cone, as can be seen in Fig. 7. The rise of the center of the bottom in the shake flask, formed during the cooling step in the production of shake flasks, is modelled in neither approach. Instead, a flat bottom with a diameter of 52.6 mm is assumed (Fig. 8B). Mean precise inner shake flask dimensions were taken from three shake flasks, after milling them to exactly half their maximal outer diameter (done by Aachener Quarzglas-Technologie Heinrich GmbH & Co.KG). Photographs of the three shake flasks are shown in the supplementary information (Fig. S2).

After the implementation of the basic flask geometry, the meshing was performed with the *blockMesh* and *snappyHexMesh* utilities, included in OpenFOAM. The finished mesh for the flask geometry with a smooth transition between torus and cone part (refer Fig. 7B) is shown in Fig. 8. During the meshing process *blockMesh* is used to create a first, basic mesh, which is then refined or *chiselled* to precisely fit the intended geometry with *snappyHexMesh*^63^. In this work a cylindrical, hexahedral mesh with a diameter slightly larger than the maximal shake flask diameter is created with *blockMesh*. Afterwards the mesh is refined with the three steps of castellation, snapping and an addition of layers with *snappyHexMesh*. During castellation all mesh cells of the cylindrical mesh, which are more than 50% outside of the flask geometry, are removed. Additionally, refinement steps, dividing one cell into eight, can be included. In this work one refinement region was applied to the entire inside of the shake flask, affecting all cells of the mesh. Furthermore, two refinement steps with the shake flask wall as the refinement surface were applied during castellation. The resulting edges of the cell refinement are clearly visible in Fig. 8. One can be seen with a diameter of 22.1 mm in Fig. 8b and one roughly 1 mm from the shake flask wall in the close-up view in Fig. 8A. The castellation creates a jagged surface at the flask wall. During snapping vertex points of those initially hexahedral cells are moved on the flask wall surface. Affected cells are no longer necessarily hexahedral afterwards. Although this step leaves a perfectly smooth surface, some, if not all, of the cells at the surface might be irregularly shaped leading to potentially poor CFD performance. Therefore, in the last step of snappyHexMesh additional layers of hexahedral cells can be added parallel to the snapped surface. In this work, three additional layers were added at the shake flask wall, as can be seen in Fig. 8A. The first layer, closest to the wall, is the thinnest layer with a thickness of roughly 140 µm, with following layers being 20% larger than the preceding layer. The generated, final mesh has about 1.4·10^6^ cells in total.

### Extraction of liquid contact lines

Liquid contact lines, as a representation of the entire liquid distribution, are extracted with the *probes* post processing function, included in OpenFOAM. The *probes* function can be used to return field values of mesh cells closest to a provided combination of cartesian coordinates. In the case of this work, the liquid volume fraction α_L_ is returned for sampled cells. Cells are sampled along the flask wall with a distance of 0.5 mm in the z-axis and 1°-steps around the central z-axis of the shake flask, leading to roughly 72,000 sampled positions. For each sampled angle, the liquid contact line is determined separately. To this end, all cells with an α_L_ value above 0.5 are considered as cells filled with water and below 0.5 with air. Descending from the top of the shake flask all changes from air filled to water filled cells and vice versa are recorded and plotted over their respective angle, leading to representations like Fig. 3. The described process is usually repeated for multiple, normal distances from the wall (Fig. 8A), as can be seen in Figs. 4 and 5. Even for the first repetition of the extraction of liquid contact lines, a distance of 50 µm is chosen to ensure sampled positions are within the mesh cells. Positions exactly on the shake flask wall might not be considered as within the meshed geometry by the *probes* function. For further repetitions, the normal distance is increased by 200 µm up to a normal distance of 1050 µm.

### Simplified mechanistic model of liquid distributions in shake flasks

For a first comparison of the liquid contact lines, the simplified, mechanistic model for liquid distributions in shake flasks by Büchs et al.^51^ was used. The model entirely neglects viscous forces and describes the shaking motion as a superposition of a translatoric and an opposing rotation of the shake flask. This allows for an approximation of the liquid distributions inside of the shake flask as an intersection of a symmetrical paraboloid and the shake flask wall geometry, where the overall shape of the paraboloid is determined by the centrifugal force. Further, the height of the origin of the paraboloid is adjusted until the volume encapsulated in the intersection of paraboloid and shake flask wall matches the desired filling volume. Lastly, it should be noted, that the simplified model uses the same basic shake flask geometry as shown in Fig. 7A, where a sharp transition between the lower torus and upper cone occurs.

### Calculation of volumetric power input from CFD simulations

To calculate the volumetric power input from CFD simulations, the relationship to the energy dissipation rate was used:

20 $$\frac{P}{{V}_{L}}=\varepsilon\cdot \rho$$

where P, V_L_ and ε are the power input in W, the liquid volume in m^3^ and the energy dissipation rate in W/kg. This approach has been used before for the simulation of shaken system in general^15–17^ and shake flasks specifically^19^. The total energy dissipation consists of the dissipation of the mean flow and dissipation caused by the turbulence. The energy dissipation of the mean flow can be calculated according to:

21 $${\varepsilon }_{m}=\frac{\eta }{\rho }|\mathbf{S}|$$

The turbulent energy dissipation can be taken from the conservation equation of turbulent kinetic energy (Eq. 9):

22 $${\varepsilon }_{t}={\beta }^{*}k\omega$$

Hence, the energy dissipation rate for the entire liquid volume can be calculated as follows:

23 $$\varepsilon =\frac{{\int }_{{V}_{L}}\left(\frac{\eta }{\rho }\cdot \left|{\text{S}}\right|+{\beta }^{*}\omega k\right)\cdot d{V}_{L}}{{V}_{L}}$$

It should be noted, that the liquid volume for a specific cell i in Eq. (23) must be calculated according to the VOF model as:

24 $${V}_{L,i}={V}_{i}\cdot {\alpha }_{L,i}$$

where V_L,i_ V_i_ and α_L,i_ are the liquid volume, total cell volume and liquid volume fraction of cell i.

### Volumetric power input correlation

Volumetric power inputs for shake flasks can be calculated from the correlation of the modified Newton number (Ne^′^) and Reynolds number (Re)^43^ by Büchs et al.^44^:

25 $${\text{Ne}}^{\prime } = 70 \cdot {\text{Re}}^{ - 1} + 25 \cdot {\text{Re}}^{ - 0.6} + 1.5 \cdot {\text{Re}}^{ - 0.2}$$

where the Reynolds number (-) and modified Newton number (-) are given as follows:

26 $${\text{Ne}}^{\prime } = \frac{P}{{\rho \cdot n^{3} \cdot d^{4} \cdot V_{L}^{\frac{1}{3}} }}$$

27 $${\text{Re}}=\frac{\rho\cdot n\cdot {d}^{2}}{\eta }$$

### Experimental data sets

Experimental data for the comparison of liquid contact lines was provided by Azizan et al.^37,38^. The data set includes shaking frequencies from 150 to 450 rpm, filling volumes from 15 to 40 mL, at a shaking diameter of 2.5 cm for a viscosity of 0.89 and 16.7 mPa∙s. The data set form Büchs et al. contains volumetric power inputs for a broad range of shaking conditions, including different shake flasks sizes, shaking frequencies, shaking diameters, filling volumes and viscosities^43,44^. A subset of the data set for 250 mL shake flasks, including shaking frequencies from 180 to 380 rpm, filling volumes of 25 and 40 mL at a shaking diameter of 2.5 cm for a viscosity of 1 and 16 mPa∙s, was used for the comparison to the CFD model.

### Supplementary Information

Supplementary Information.

## Data Availability

The datasets generated during and/or analysed during the current study are available from the corresponding author on reasonable request.

## List of symbols

CFD: Computational fluid dynamics
STR: Stirred tank reactor
VOF: Volume of fluid
k_L_a: Volumetric gas–liquid mass transfer rate (h^−1^ or s^−1^)
d_0_: Shaking diameter (cm or m)
n: Shaking frequency (min^−1^ or s^−1^)
t: Time (s)
d: Largest inner flask diameter (mm or m)
V_L_: Filling volume (mL or L)
ρ: Density (kg/m^3^)
P: Pressure (Pa)
**g**: Gravitational acceleration (m/s^2^)
P: Power input (W or kg m^2^/s^3^)
Ne^′^: Modified Newton number (-)
Re: Reynolds number (-)
σ: Surface tension constant (N/m)
F_σ_: Surface tension (N/m)
ε: Energy dissipation rate (W/kg or m^2^/s^3^)
**U**: Velocity vector (m/s)
**S**: Strain rate tensor (s)
α: Volume fraction (-)
τ: Viscous stress (Pa)
τ_t_: Turbulent stress (Pa)
η: Dynamic viscosity (Pa∙s)
κ: Approximation of the curvature at the interface (-)
$\overrightarrow{\omega }$: Angular velocity (rad/s)
k: Turbulent kinetic energy (m^2^/s^2^)
ω: Specific energy dissipation rate (s^−1^)
