# Supplementary material for: Validation of computational fluid dynamics of shake flask experiments at moderate viscosity by liquid distributions and volumetric power inputs
Source: Sci Rep. 2024 Feb 13;14:3658. doi: 10.1038/s41598-024-53980-7 (PMC10864319; doi:10.1038/s41598-024-53980-7)
Supplement: Supplementary file 1 — Supplementary Information. [file 41598_2024_53980_MOESM1_ESM.docx]

# Supplementary data:

**Computational Fluid Dynamics for shake flask experiments at moderate viscosity, validated by liquid distributions and volumetric power inputs**

Carl Dinter, Andreas Gumprecht, Matthias Alexander Menze, Amizon Azizan, Paul-Joachim Niehoff, Sven Hansen and Jochen Büchs

Table S1: Material parameters for the CFD simulations.

Parameters are taken from the VDI Heat Atlas.

| σ_water,30°C_ | 70 mN/m |
| --- | --- |
| ρ_water,25°C_ | 997 kg/m^3^ |
| ρ_water,37°C_ | 993 kg/m^3^ |
| η_water,25°C_ | 0.89 mPa·s |
| η_water,37°C_ | 0.69 mPa·s |
| ρ_air,30°C_ | 1.2 kg/m^3^ |
| η_air,30°C_ | 0.02 mPa·s |


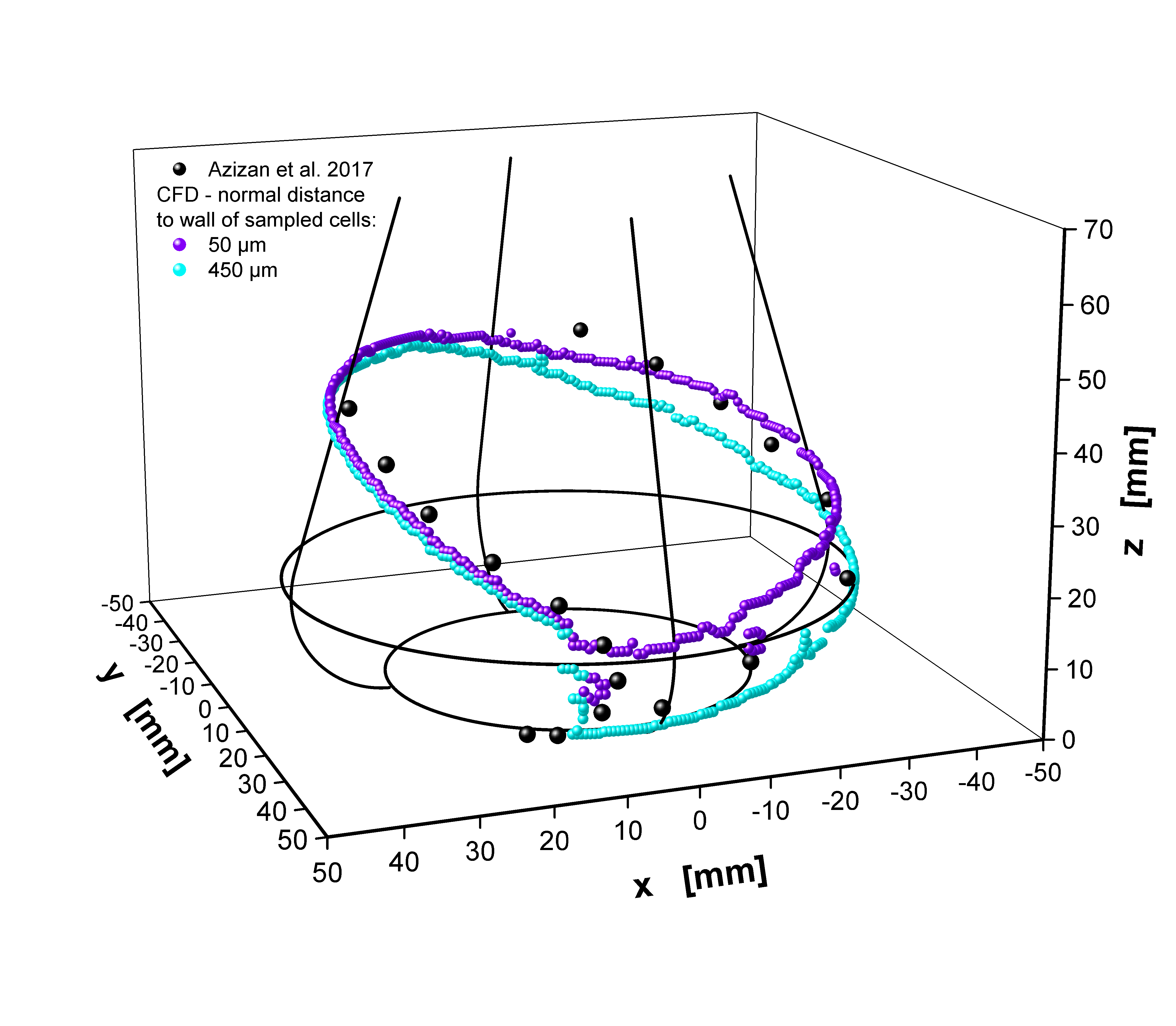


Fig. S 1: 3D-representation of the comparison of liquid contact lines from CFD and experimental data (Azizan et al. 2017)

Depicted is the same simulation as in Fig. 5A, only as a 3D-representation for distances of 50 to 450 µm normal to the shake flask wall to exclude the liquid film (see Fig. 8A). The figure becomes incomprehensible for more than two normal distances. Simulated conditions: Viscosity (η) = 0.69 mPa·s, shaking diameter (d_0_) = 2.5 cm, filling volume (V_L_) = 40 mL, shaking frequency (n) = 250 rpm, surface tension (σ) = 70 mN/m, contact angle (θ) = 20°, temperature (T) = 25°C


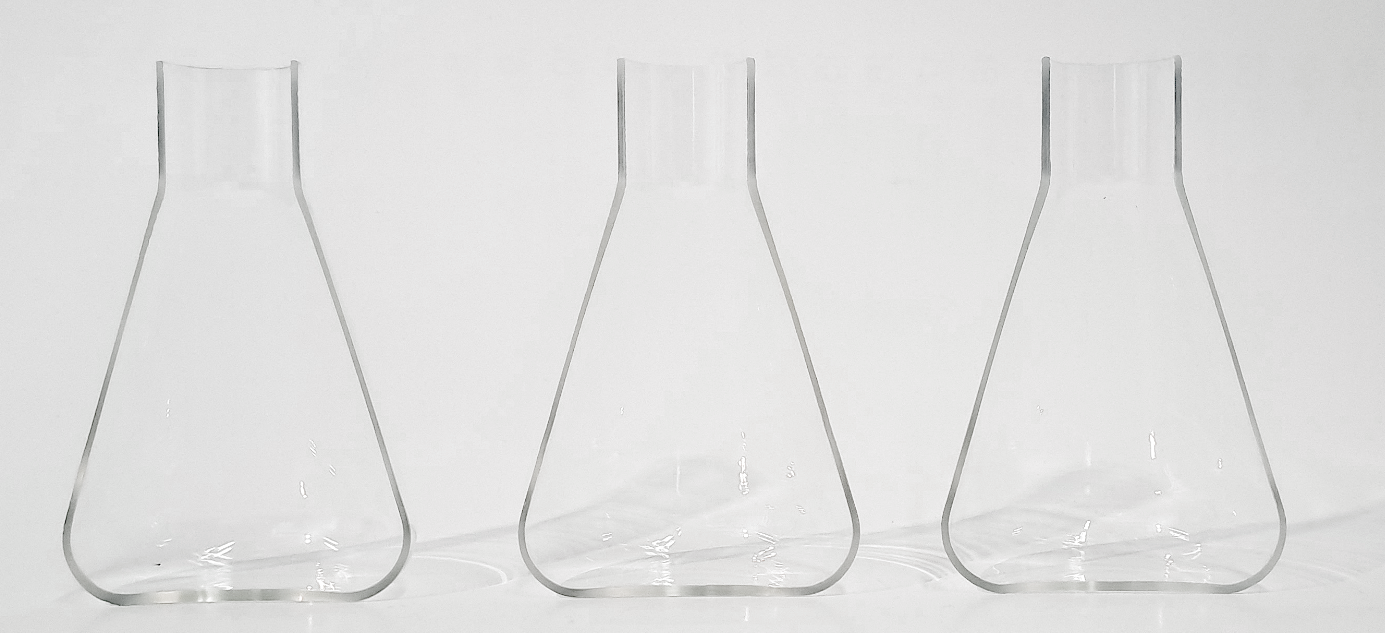


Fig. S2: Shake flasks milled to half their maximal outer diameter to evaluate the exact geometry of the flasks.

Front view of milled down shake flasks. Three shake flasks of the same type and from same manufacturer were used as replicates. Inner measurements of the flask geometry provided in the main manuscript are mean values from the three shown flasks. Shake flasks were milled down by Aachener Quarzglas-Technologie Heinrich GmbH & Co.KG. Results are specified in Fig. 7 and 8.


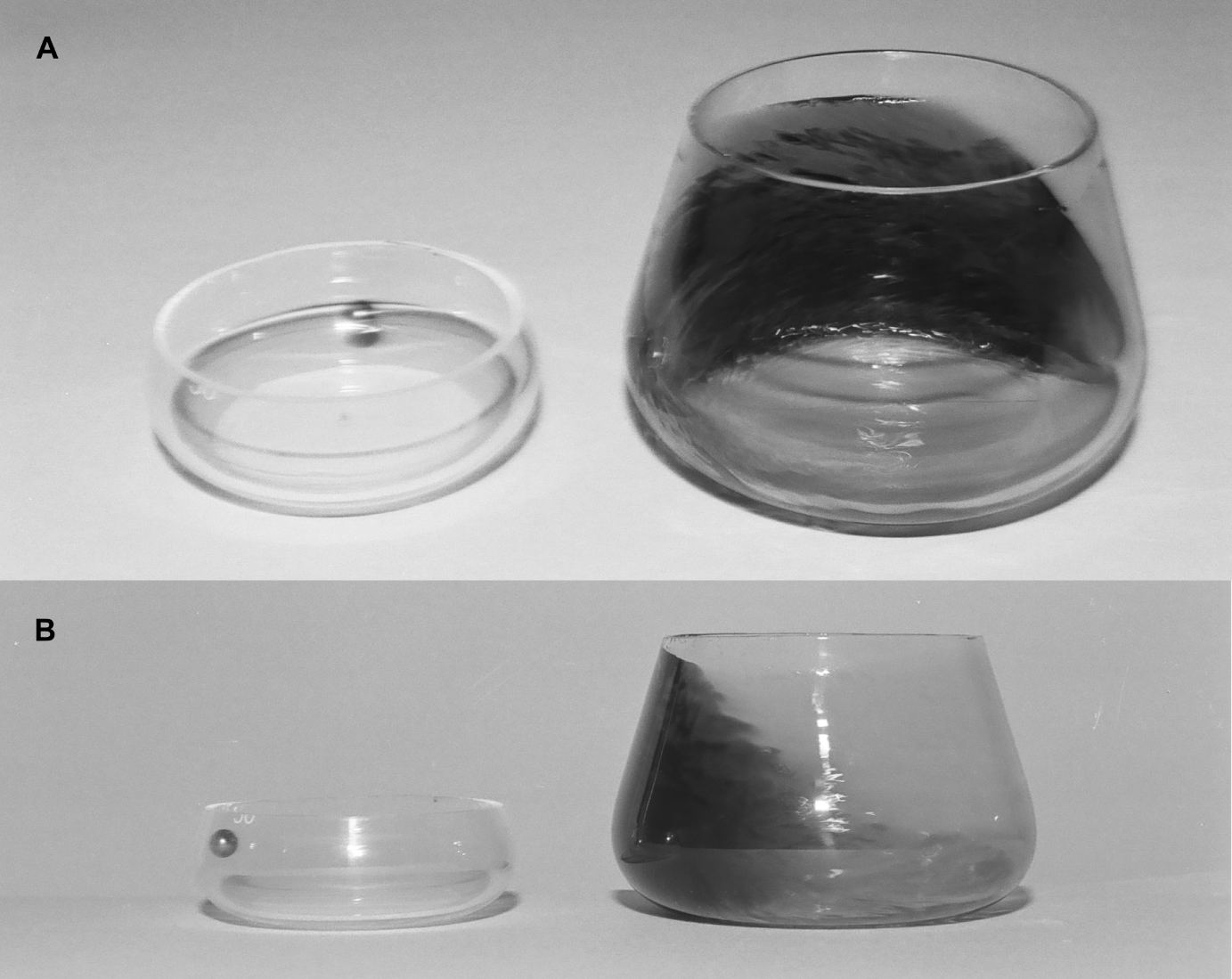


Fig. S3: Photograph of the liquid distribution at a shaking frequency of 450 rpm, viscosity of 1 mPa·s, shaking diameter of 2.5 cm and filling volume of 25 mL.

In (A) a photograph of the rotating bulk liquid in line with the centrifugal force is shown. The photograph in (B) is taken perpendicular to the centrifugal force, focusing on the tail region, following the rotating bulk liquid. The small metal balls on the left of (A) and (B) are included to show the direction of the centrifugal force. The formed liquid film can be seen in (B) as a light shadow up to the maximal liquid height. Contrary to all previous images, experimental results and CFD simulations, the shaking motion in these images is counter-clockwise.


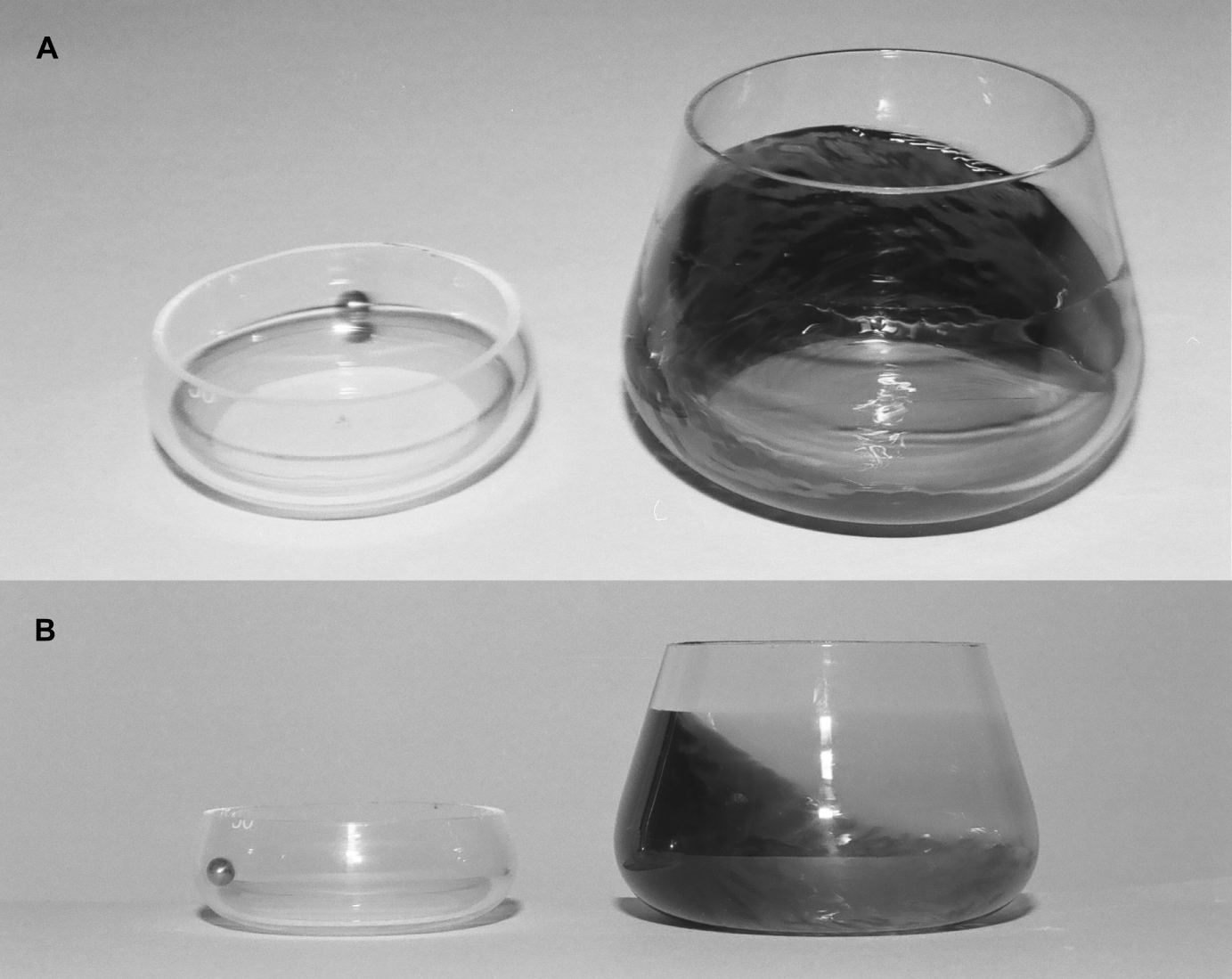


Fig. S4: Photograph of the liquid distribution at a shaking frequency of 300 rpm, viscosity of 1 mPa·s, shaking diameter of 2.5 cm and filling volume of 25 mL.

In (A) a photograph of the rotating bulk liquid in line with the centrifugal force is shown. The photograph in (B) is taken perpendicular to the centrifugal force, focusing on the tail region, following the rotating bulk liquid. The small metal balls on the left of (A) and (B) are included to show the direction of the centrifugal force. The formed liquid film can be seen in (B) as a light shadow up to the maximal liquid height. Contrary to all previous images, experimental results and CFD simulations, the shaking motion in these images is counter-clockwise.

*
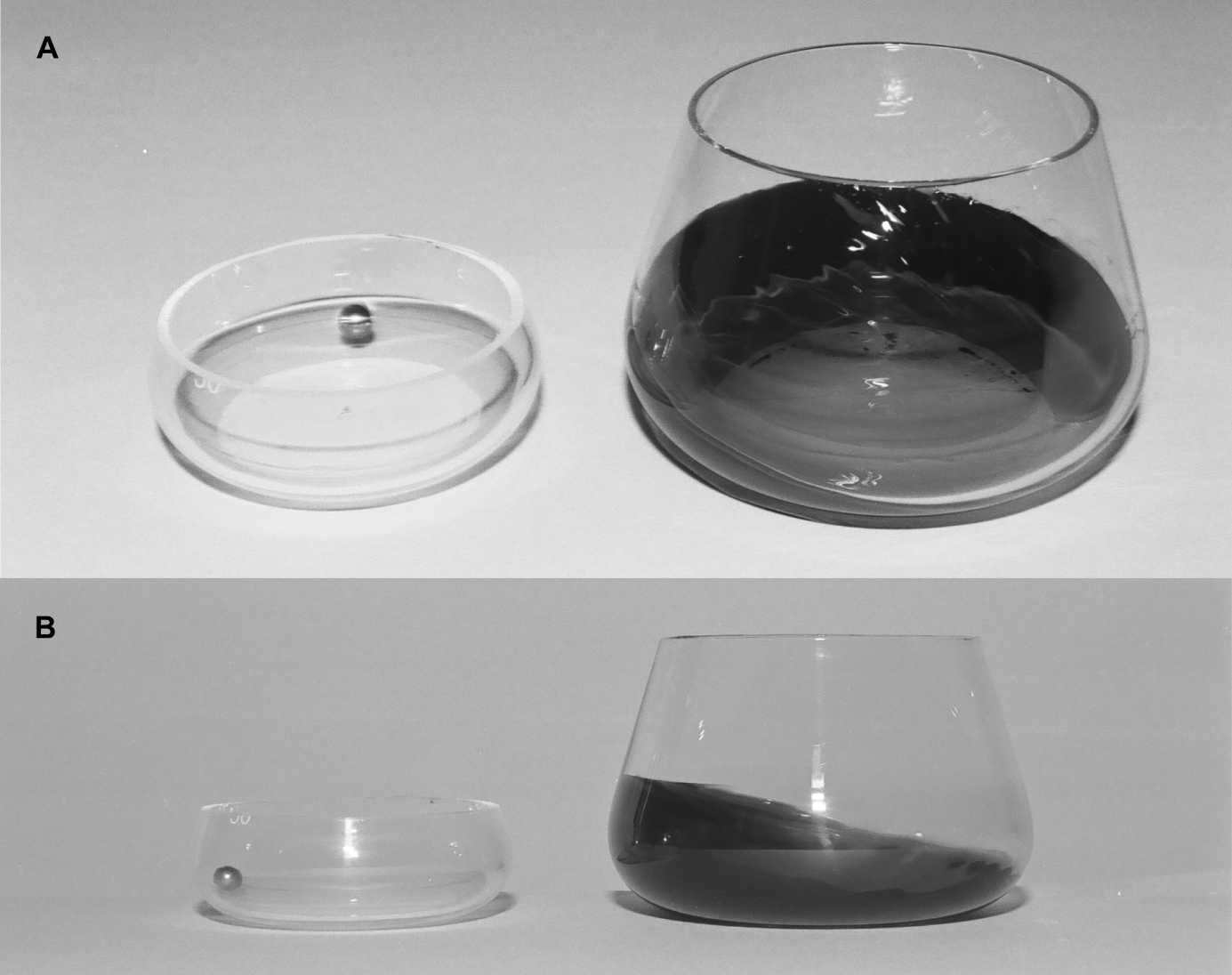
*

Fig. S5: Photograph of the liquid distribution at a shaking frequency of 150 rpm, viscosity of 1 mPa·s, shaking diameter of 2.5 cm and filling volume of 25 mL.

In (A) a photograph of the rotating bulk liquid in line with the centrifugal force is shown. The photograph in (B) is taken perpendicular to the centrifugal force, focusing on the tail region, following the rotating bulk liquid. The small metal balls on the left of (A) and (B) are included to show the direction of the centrifugal force. Contrary to all previous images, experimental results and CFD simulations, the shaking motion in these images is counter-clockwise.


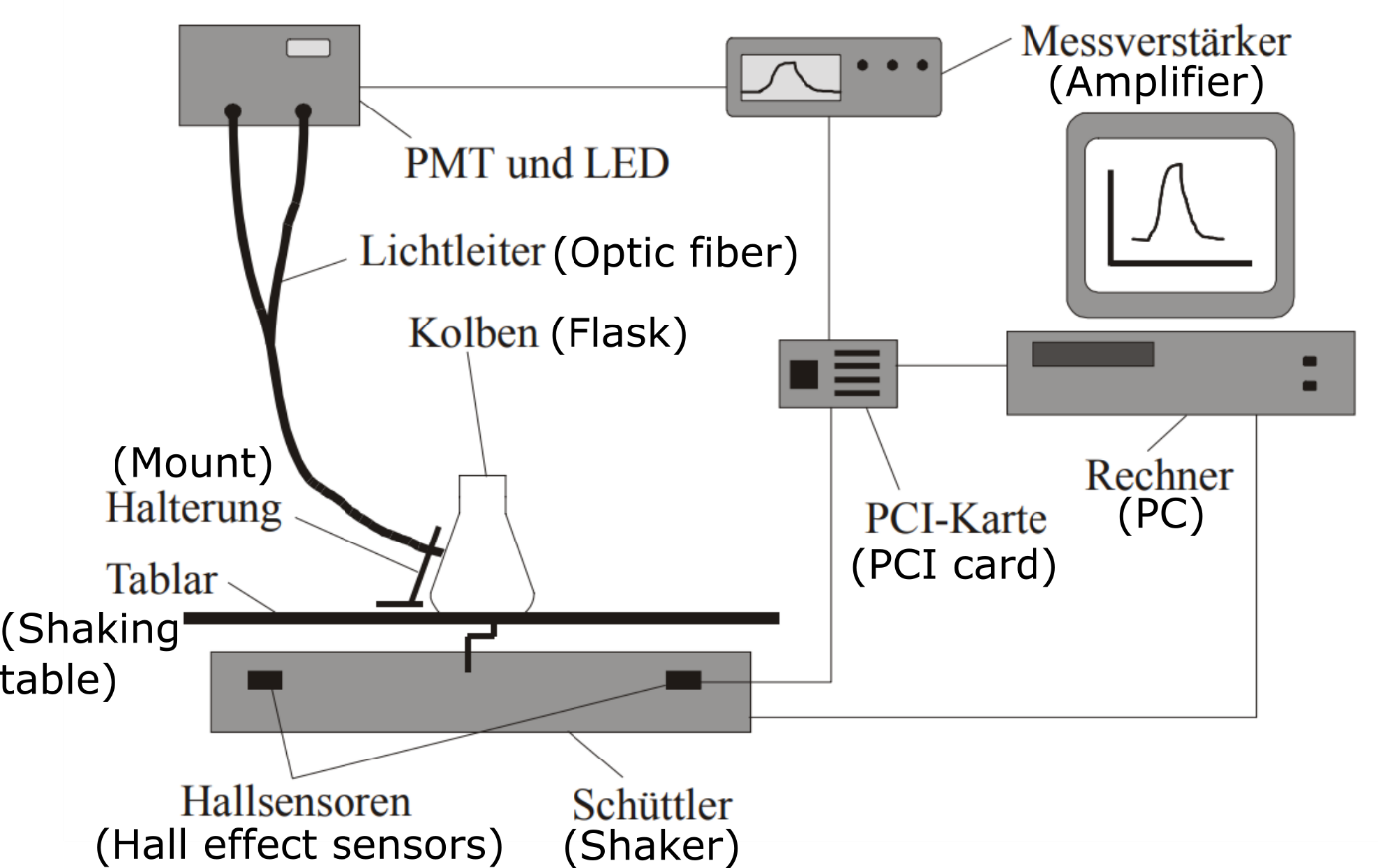


Fig. S6: Schematic of the setup for liquid thickness measurements (taken from Hermann 2001).

The figure is taken from the dissertation of Hermann. Translations added in brackets. The setup was used to perform liquid thickness measurements in shake flasks. An optical fiber is mounted next to a shake flask, placed on a shaker table. Half of the optical fiber is connected to a blue LED (350 – 500 nm) for excitation of fluorescence and the other half to a photomultiplier (PMT) to record the fluorescence emission (above 570 nm). The signal form the PMT was transmitted through an amplifier and recorded on a computer. Hall effect sensors were installed within the shaker to monitor the position of the shaker and the direction of centrifugal force. The measurement frequency was 5000 Hz.


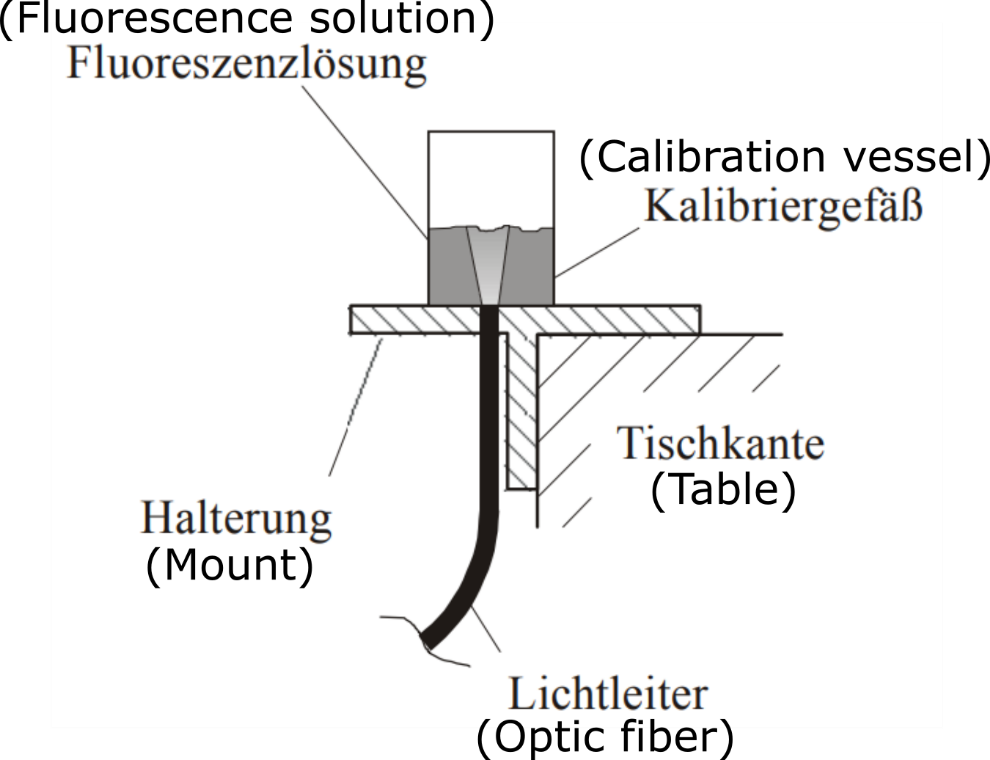


Fig. S7: Schematic of setup for the calibration of liquid thickness measurements (taken form Hermann 2001).

The figure is taken from the dissertation of Hermann. Translations added in brackets. The setup was used to record a calibration, to convert recorded fluorescence intensities (Fig. S6) to liquid thickness. A custom plexiglass well was constructed and placed above the optical fiber of the fluorescence measurement system (Fig. S6). For the calibration the volume, hence, height of the fluorescent solution was incrementally increased and the resulting fluorescence intensity measured. With this data a calibration curve was created.


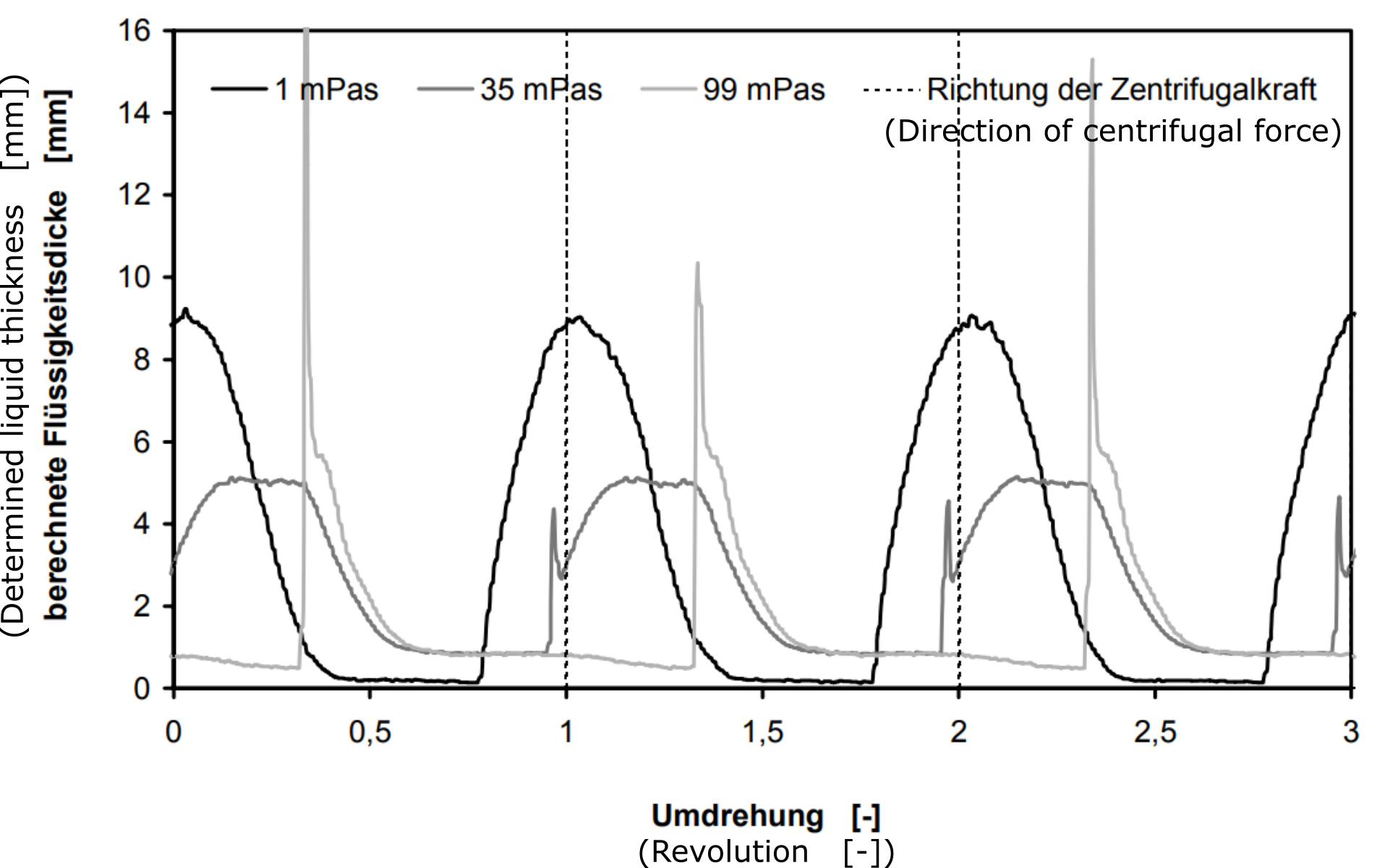


Fig. S8: Liquid thickness in shake flasks for three viscosities based on fluorescence measurements (taken from Hermann 2001).

The figure is taken from the dissertation of Hermann. Translations added in brackets. Measurements were performed with the setup shown in Fig. S6 and calibrated according to Fig. S7. The calculated liquid thickness (y-axis) is plotted over three full rotations of the shake flask (x-axis) for three different viscosities. The dashed vertical lines indicate the direction of the centrifugal force. At zero revolutions the bulk liquid is in front of the sensor for 1 mPa·s. At this point the liquid thickness of the bulk liquid is determined to be roughly 9 mm. After half a rotation the bulk liquid has passed the sensor and the liquid thickness of the film is measured with about 50 µm. At a viscosity of 35 and 99 mPa·s, the liquid film starts similar in liquid thickness with about 800 µm. At 35 mPa·s, the film stays at that thickness until the bulk liquid moves in front of the sensor again. At 99 mPa·s, the film thickness decreases further to about 500 µm. The spike in the liquid thickness, when the bulk liquid arrives at the sensor (measured fluorescence intensity) at 35 and 99 mPa·s is explained by Hermann with possible reflection effects in the leading edge of the bulk liquid. Experimental conditions: Viscosity (η) = 1, 35, 99 mPa·s, shaking diameter (d_0_) = 2.5 cm, filling volume (V_L_) = 25 mL, shaking frequency (n) = 300 rpm, 2.5 µm fluorescein, fluorescence sensor height = 20.6 mm.


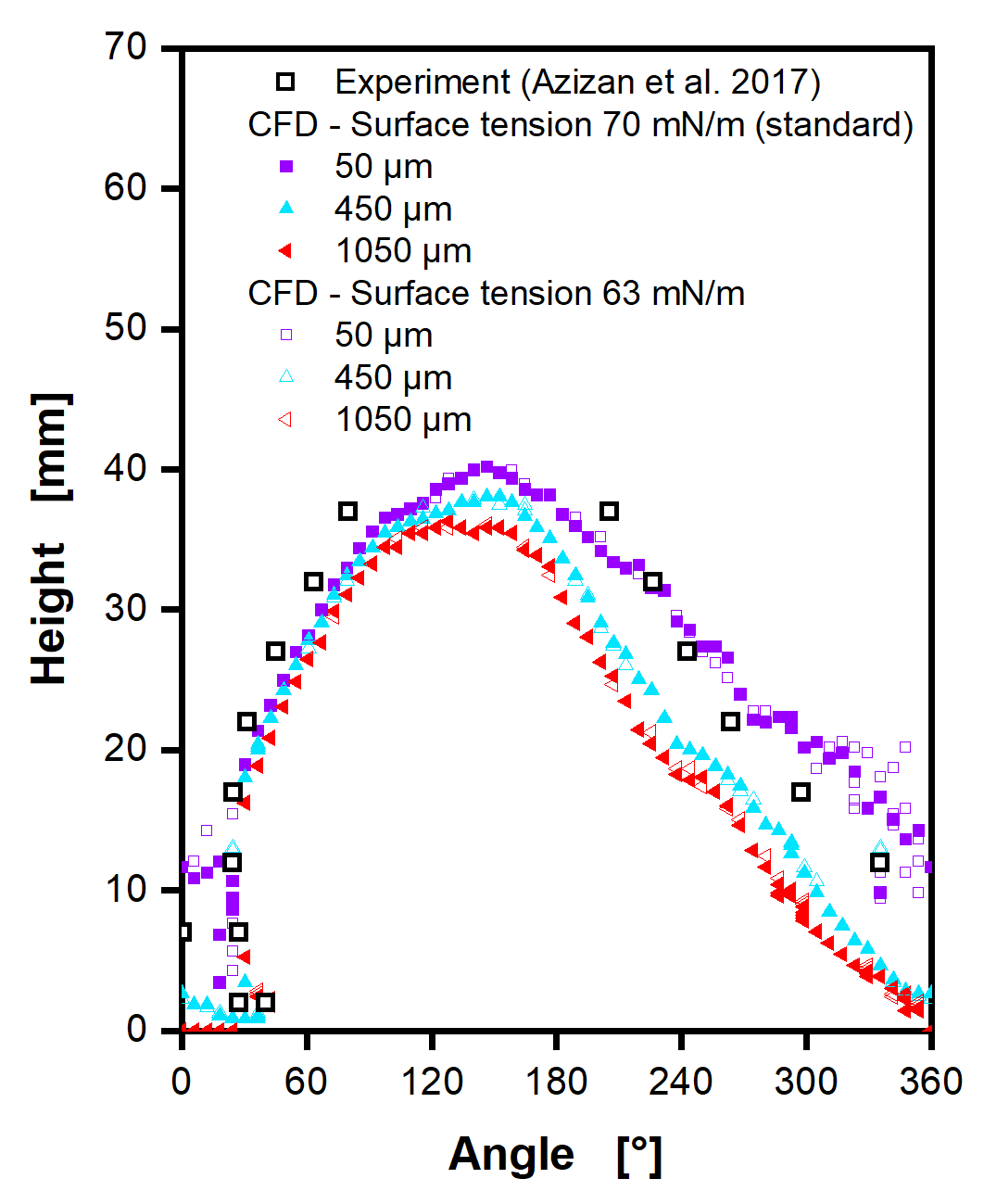


Fig. S9: Impact of the assumed surface tension on the liquid contact line calculated by the CFD model, compared to experimental data (Azizan et al. 2017)

The liquid contact lines are shown in mm, viewed from the center of the shake flask, rotating around the z-axis (see Fig. 1C). Liquid contact lines from CFD calculations were extracted at multiple distances from 50 to 1050 µm normal to the shake flask wall to exclude the liquid film (see Fig. 8A). Simulated conditions: Viscosity (η) = 0.69 mPa·s, shaking diameter (d_0_) = 2.5 cm, filling volume (V_L_) = 30 mL, shaking frequency (n) = 250 rpm, surface tension (σ) = 70 mN/m and 63 mN/m, contact angle (θ) = 20°, temperature (T) = 37°C


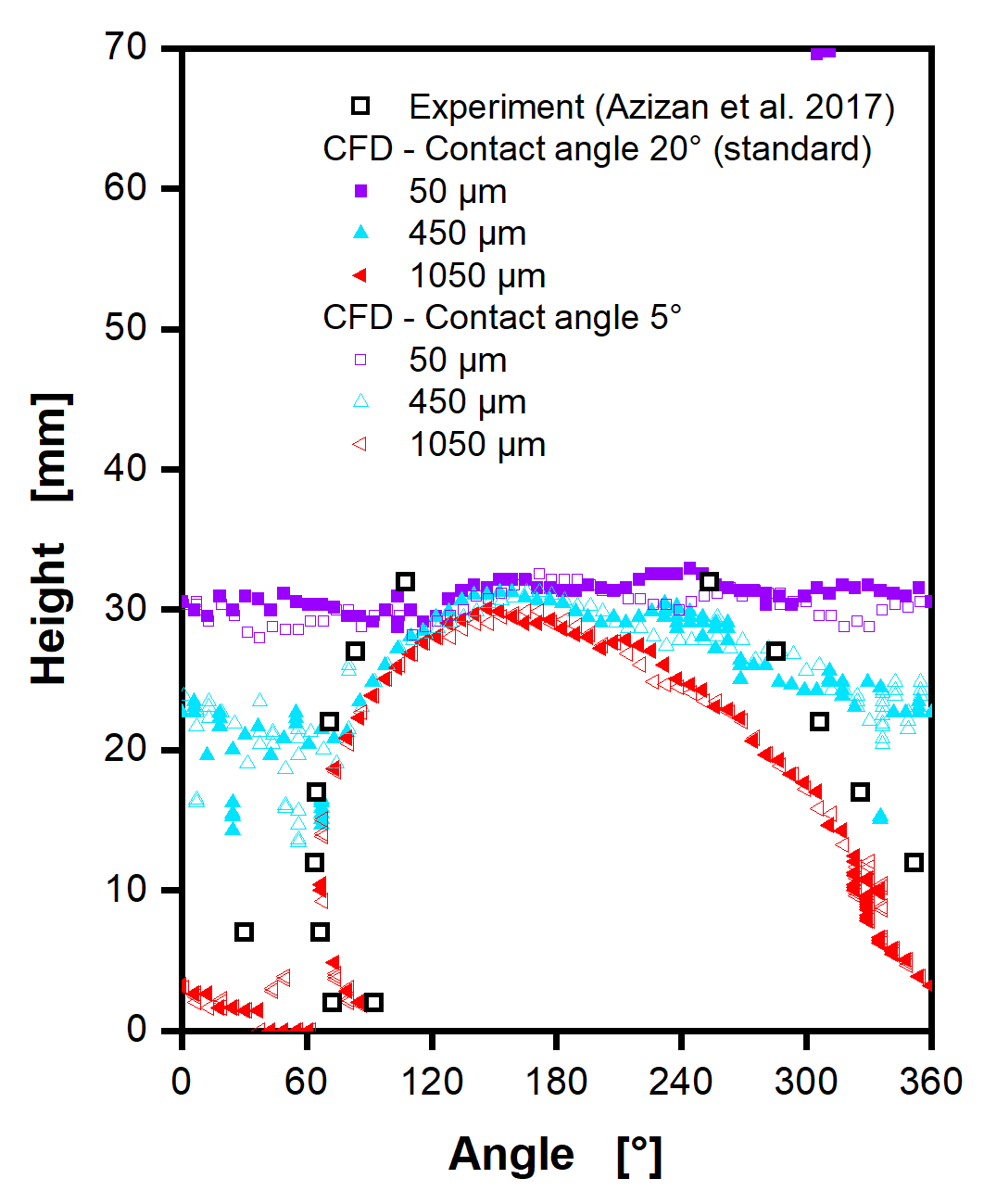


Fig. S10: Impact of the assumed contact angle on the liquid contact line calculated by the CFD model, compared to experimental data (Azizan et al. 2017)

The liquid contact lines are shown in mm, viewed from the center of the shake flask, rotating around the z-axis (see Fig. 1C). Liquid contact lines from CFD calculations were extracted at multiple distances from 50 to 1050 µm normal to the shake flask wall to exclude the liquid film (see Fig. 8A). Simulated conditions: Viscosity (η) = 16.7 mPa·s, shaking diameter (d_0_) = 2.5 cm, filling volume (V_L_) = 30 mL, shaking frequency (n) = 250 rpm, surface tension (σ) = 70 mN/m, contact angle (θ) = 20° and 5°, temperature (T) = 25°C


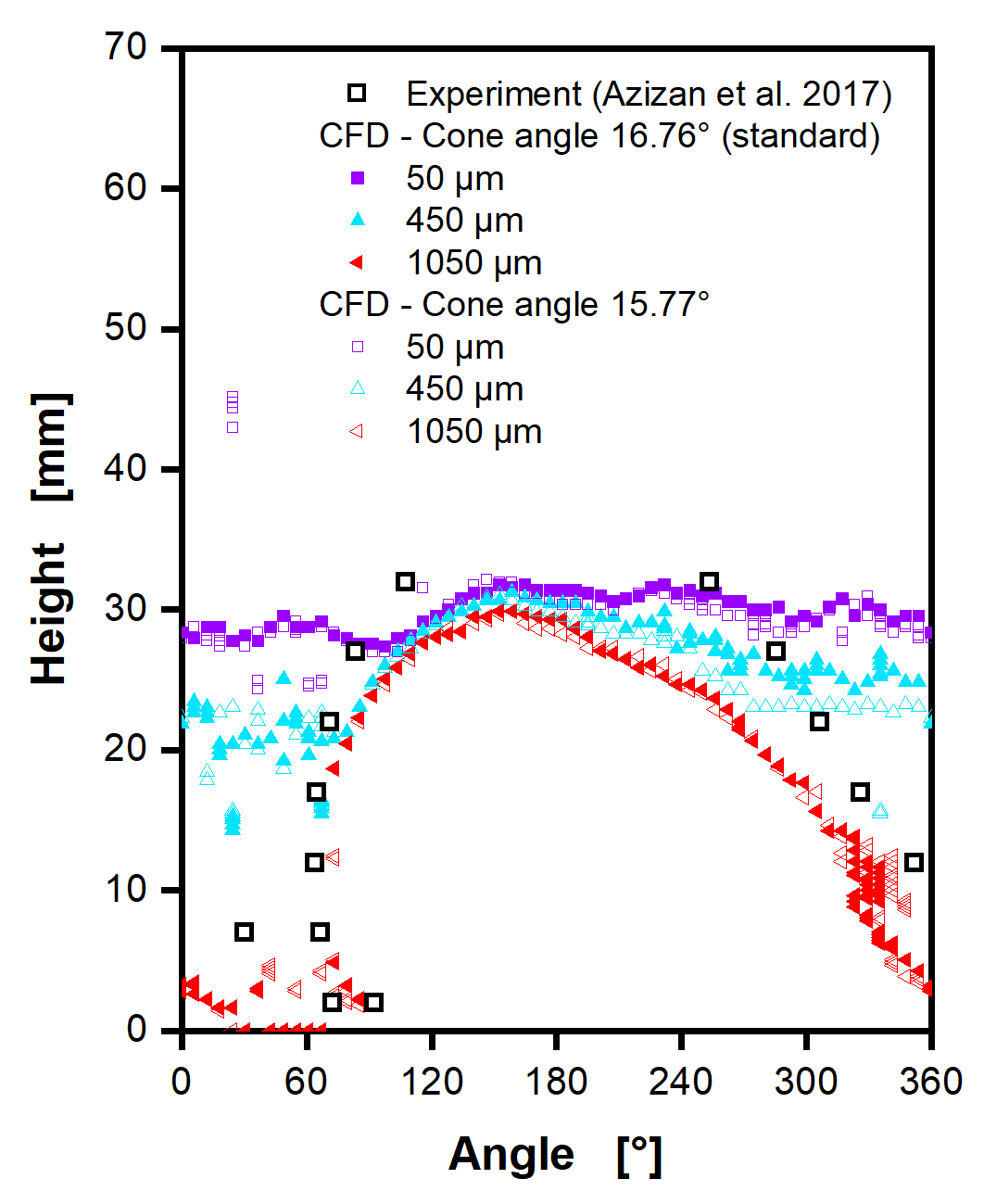


Fig. S11: Impact of the cone angle on the liquid contact line calculated by the CFD model, compared to experimental data (Azizan et al. 2017)

The liquid contact lines are shown in mm, viewed from the center of the shake flask, rotating around the z-axis (see Fig. 1C). Liquid contact lines from CFD calculations were extracted at multiple distances from 50 to 1050 µm normal to the shake flask wall to exclude the liquid film (see Fig. 8A). Simulated conditions: Viscosity (η) = 16.7 mPa·s, shaking diameter (d_0_) = 2.5 cm, filling volume (V_L_) = 30 mL, shaking frequency (n) = 250 rpm, surface tension (σ) = 70 mN/m, contact angle (θ) = 20°, temperature (T) = 25°C


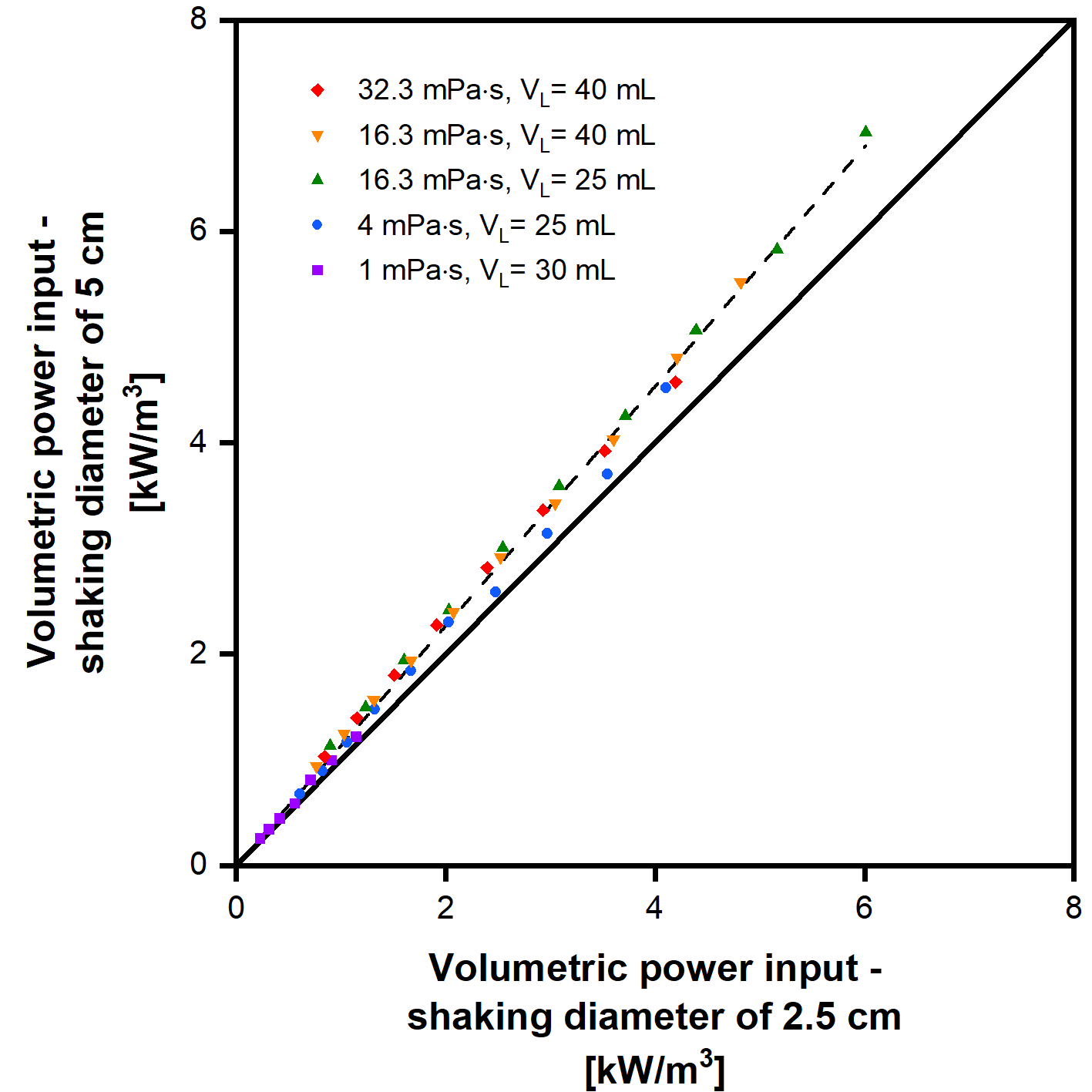


Fig. S 12: Parity plot of experimentally determined volumetric power inputs from Büchs et al. 2000 at a shaking diameter of 2.5 and 5cm.

The black bold line indicates parity and the dashed black line a linear fit of the data. The linear fit indicates roughly 13% greater volumetric power inputs at a shaking diameter of 5 cm. Experimental conditions: Viscosity (η) = 1 – 32.3 mPa·s, shaking diameter (d_0_) = 2.5 and 5 cm, filling volume (V_L_) = 25 - 40 mL, shaking frequency (n) = 180 - 360 rpm
